# Evaluation of vector system in *Saccharopolyspora erythraea* and construction of new replicative vector

**DOI:** 10.1007/s00253-026-13858-2

**Published:** 2026-05-14

**Authors:** Yana Nohach, Markiyan Samborskyy, Anja Palusczak, Andriy Luzhetskyy, Victor Fedorenko, Yuriy Rebets

**Affiliations:** 1 German-Ukrainian Core of Excellence in Natural Products Research (CENtR), Zelena Street 20, Lviv, 79005 Ukraine; 2 Department of Genetics and Biotechnology, Ivan Franko National University of Lviv, Hrushevsky Street 4, Lviv, 79005 Ukraine; 3 Explogen LLC, Volodymyra Velykogo Street 16, Lviv, 79053 Ukraine; 4 Pharmazeutische Biotechnologie, Universität des Saarlandes, Campus, Geb. C2.3, 66123 Saarbrücken, Germany

**Keywords:** *Saccharopolyspora*, Actinobacteria, Replicative vector, Integrative vector, Biotechnology, Erythromycin

## Abstract

**Abstract:**

*Saccharopolyspora erythraea* is an industrially important actinobacteria and a model in polyketide biosynthesis studies. However, its genetic toolbox remains limited. Here, we evaluated the properties of integrative and replicative vectors in *Sacch. erythraea* DSM40517. Three actinophage-based integrative vectors (φC31, φBT1, VWB) were efficiently transferred into *Sacch. erythraea*. The *attB* sites for these vector systems were identified. In case of φBT1- and VWB-based vectors, unique integration sites exists within the chromosome of the strain. In case of pSET152 (φC31), at least four different loci were identified with different efficiency of recombination. Our data revealed that all three integrative vectors are excised from the chromosome of the strain with varying frequencies, highlighting the need for selective pressures for their stable maintenance. Commonly used replicative vectors with *Streptomyces* replicons (pIJ101 and pSG5) have low transfer efficiency in *Sacch. erythraea*. To solve this problem, we constructed a shuttle vector pYS191 based on the replicon of plasmid pJV1. This vector was efficiently transferred into *Sacch. erythraea* and other actinobacteria including *Streptomyces*, *Saccharothrix* and *Couchioplanes* species*.* pYS191 is maintained at a low copy number under selective conditions, and is rapidly lost without antibiotic pressure. Neither integrative nor replicative vectors significantly affected erythromycin production. This study provides a comprehensive assessment of vector system in *Sacch. erythraea* and reports a new replicative vector based on rarely exploited pJV1 replicon for transient gene expression and genome engineering. These results expand the genetic toolbox for *Saccharopolyspora* and facilitate its use in natural products research and industrial biotechnology.

**Key points:**

• *Actinobacterial integrative vectors are suited for gene cloning in Sacch. erythraea.*

• *attB sites for three integrative vectors identified within Sacch. erythraea genome.*

• *A new replicative vector pYS191 for use in Sacch. erythraea was constructed.*

**Supplementary Information:**

The online version contains supplementary material available at 10.1007/s00253-026-13858-2.

## Introduction

The genus *Saccharopolyspora* is currently accounting for 40 species with validly published names (Sayed et al. 2020). The genus was first described by Lacey and Goodfellow in 1975 and revised later by Korn-Wendisch et al. (1989) (Korn-Wendisch et al. 1989). It is a member of the *Pseudonocardiaceae* family which also includes *Pseudonocardia* and *Saccharomonospora* genera. *Saccharopolyspora* species are widely spread in nature and could be found in soil, marine sediments, marine invertebrates, plants and clinical samples (Sayed et al. 2020). They are known for the production of diverse secondary metabolites, including macrolide antibiotic erythromycin A produced by *Sacch. erythraea* and insecticide spinosyn produced by *Sacch. spinosa*.

*Streptomyces erythraeus (Actinomyces erythreus)* was originally described by Waksman and later reclassified into the *Saccharopolyspora erythraea* (Labeda 1987). The strain is a producer of the important antibiotic erythromycin which not only has medical application but also served for many years as a model for studying the polyketides assembly by type I polyketide synthase (Leadlay et al. 1993). In 2007, the complete genome sequencing of *Sacch. erythraea* NRRL23338 was reported revealing the presence of 25 gene clusters involved in the biosynthesis of secondary metabolites (Oliynyk et al. 2007). Furthermore, *Sacch. erythraea* and its derivatives often are considered as a potent non-Streptomyces host strain for heterologous production of bacterial natural products (Baltz 2010). In fact, *Sacch. erythraea* is the most studied species among all members of the *Pseudonocardiaceae* family. Despite being a model organism for the entire family and the long-standing history of research, surprisingly little is known about the behaviour of cloning vectors in this species.

The integrative vectors are considered to be highly efficient and such that could be stably maintained without antibiotic pressure (Rebets et al. 2017). The most popular actinobacterial integrative vectors are based on site-specific recombination systems of φC31, φBT1 and VWB actinophages (Kieser et al. 2000). These vectors integrate into the genome by the recombination between phage *attP* site and *attB* locus of chromosome. The *attB* sites are highly specific for each phage, and thus the corresponding integrative vectors are always inserted at the same location within the genome. However, the attempts to identify the φC31 attachment site in *Sacch. erythraea* led to the conclusion that the strain lacks the consensus *Streptomyces*-like *attB* sequence, and pSET152 integrates into seven distinct loci considered to be a pseudo-*attB* (Rodriguez et al. 2003). This prompted the engineering of *Sacch. erythraea* by inserting the *Streptomyces* φC31 *attB* sequence into the genome in order to facilitate the stable maintenance of the genetic constructs (Rodriguez et al. 2003; Wu et al. 2011). A similar approach was undertaken to construct *Sacch. erythraea* strains suitable for the φBT1 actinophage-based vectors like pRT801 and its derivatives (Lu et al. 2020). To our knowledge, no attempts on direct transfer of pRT801 and other φBT1-based vectors into *Sacch. erythraea* were done due to the generally accepted consideration that the native *attB* site of this actinophage is absent from the chromosome of the strain. Finally, the VWB actinophage, originally isolated from *Streptomyces venezuelae*, serves as a popular alternative to φC31 and φBT1 integrative systems with several vectors widely used (pVWB, pSOK804, pTOS(z)) (Van Mellaert et al. 1998). Some of these vectors were also used for gene expression in *Sacch. erythraea*; however, their properties and influence on the strain behaviour are not studied (Gutacker et al. 2020).

On the other hand, the commonly used replicative vectors are poorly suited for stable gene expression in *Sacch. erythraea* (Yamamoto et al. 1986)*.* Currently, three replicons are most often used in actinobacterial replicative vectors: the high copy number pIJ101 from *S. lividans* ISP5434 (Kieser et al. 1982); the moderate copy number temperature sensitive pSG5 from *S. ghanaensis* (Muth et al. 1988) and the low copy number SCP2 from *S. coelicolor* A3(2) (Bibb et al. 1977). All three groups of vectors are difficult to introduce into the *Sacch. erythraea* either by transformation or conjugation (Wang et al. 2008; Yamamoto et al. 1986). The pIJ101-based vectors are rapidly lost by the strain even under the antibiotic pressure (Weber and Losick 1988). The SCP2 replicon-based vectors are also poorly maintained in *Sacch. erythraea* and were reported to undergo structural rearrangements (Yamamoto et al. 1986). Despite this, the pIJ101 and pSG5-based vectors are being used for transient gene expression in *Sacch. erythraea*, primarily CRISPR-Cas genome editing (Liu et al. 2018; Mo et al. 2019).

The vectors based on the replicon of the pJV1 plasmid isolated from *Streptomyces phaeochromogenes* were reported to be efficiently transformed and stably maintained in *Sacch. erythraea* (Servin-Gonzalez et al. 1995; Yamamoto et al. 1986). Several pJV1 vectors were constructed, including pWOR series and pWHM4 (Bailey et al. 1986; Vara et al. 1989). However, all of them have a number of significant drawbacks: inconvenient selection markers, lack of conjugation transfer function, accessibility, etc. Here we report a systematic characterisation of commonly used integrative and replicative vectors in *Sacch. erythraea*, as well as the construction of a novel conjugative shuttle vector that is stably maintained in this important industrial species.

## Materials and methods

### Strains, plasmids and cultivation conditions

The bacterial strains and plasmids used in this work are listed in Tables S1 and S2 respectively. *Sacch. erythraea* DSM40517 strain was grown on GYM agar (Kieser et al. 2000). *S. albidoflavus* Del14 strain was grown on mannitol soy flour agar (MS agar) (Kieser et al. 2000). For biomass accumulation, all actinobacteria were cultivated in liquid tryptic soy broth (TSB) medium (Condalab, Spain). For erythromycin production, liquid EFM medium (40 g corn starch, 30 g soybean flour, 30 g dextrin, 2 g (NH_4_)_2_SO_4_, 10 g soybean oil, 6 g CaCO_3_, 1 L of distilled water, pH 7.2) was used (Lu et al. 2020). For conjugation, the oat-agar medium was used (36 g oat flakes, 20 g agar, 1 L of tap water, pH7.2). Actinobacteria were grown at 30 °C. *Escherichia coli* GB2005 was used as a host in cloning procedures (Fu et al. 2008). *E. coli* WM6026 served as a donor in intergeneric conjugation (Circello et al. 2010). *E. coli* strains were cultivated in liquid or on solid Luria–Bertani (LB) medium at 37 °C (Condalab, Spain). The following antibiotics were added to the medium when required: fosfomycin (100 μg/mL) (Alfa Aesar, UK), ampicillin (100 μg/mL) (Carl Roth, Germany), apramycin (100 μg/mL or 50 μg/mL) (Thermo Fisher Scientific, USA), hygromycin (50 μg/mL) (VWR, USA), thiostrepton (50 μg/mL) (EMD Millipore, USA), X-gal (50 μg/mL) (Carl Roth, Germany) and IPTG (100 μg/mL) (Carl Roth, Germany).

### Genetic manipulation

DNA isolation and manipulation and *E. coli* transformation were performed according to standard protocol (Green and Sambrook 2012; Kieser et al. 2000). pWOR191 plasmids (gift of John Innes Centre, Norwich Research Park, Norwich, NR4 7UH, UK) were isolated from *Streptomyces lividans* 3215 using modified alkaline lysis protocol. The lysis step was performed in a TE buffer supplemented with 5 mg/ml of lysozyme (Carl Roth, Germany) at 37 °C for 30 min following the standard procedure (Kieser et al. 2000). Dream Taq polymerase (Thermo Fisher Scientific, USA) was used in cloning experiments, colony PCR screening and strain verification. DNA fragments were purified from 1.2% agarose gels using the QIAquick Gel Extraction Kit (Qiagen, Germany). Restriction enzymes and ligase were used according to manufacturer recommendations (Thermo Fisher Scientific, USA, New England Biolabs, USA). Primers used in this study were purchased from GenScript Biotech B.V. (Netherlands) and are listed in Table S3. Genomic DNA was extracted with the NucleoSpin Microbial DNA Kit (Macherey–Nagel, Germany). Genomic DNA was sequenced with DNBseq (2 × 150 nucleotide) at BGI Tech Solutions (Poland). All bioinformatic manipulations were performed using the Geneious Prime v. e2024.0.7 (Dotmatics, USA).

### Intergeneric conjugation frequency evaluation

*E. coli* WM6026 carrying studied plasmids served as a donor. *Sacch. erythraea* DSM 40517 was grown on GYM agar for 14 days and *S. albidoflavus* Del14 on MS agar for 5 days to achieve sporulation. Spore suspensions were prepared in TSB medium, and spore titre was determined by serial dilutions plating. One milliliter of spore suspension was heat-shocked for 10 min at 55 °C for *Sacch. erythraea* and 42 °C for *S. albidoflavus*. The suspension of cells of overnight culture of *E. coli* donor strain was mixed with spores thoroughly by pipetting and spread on the oat meal agar plates. After incubation for 20 h for *Sacch. erythraea* and 8 h for *S. albidoflavus*, each plate was overlaid with fosfomycin (100 μg/mL) in combination with the selective antibiotic (100 μg/mL) specific for each plasmid. The modifications of protocol were applied when needed—the time and temperature of heat-shock, media used for mating, duration of incubation before antibiotic overlay. Transconjugants were counted on the 14th day of incubation for *Sacch. erythraea* and on the 5th day for *S. albidoflavus*. Vector conjugation frequency was calculated as a ratio of the number of exconjugants and the number of recipient cells in 1 mL of spore suspension. The experiments were triplicated, and the average efficiency of plasmid transfer was calculated accordingly.

### Identification of vectors integration sites in the *Sacch. erythraea* chromosome

Genomic DNA of *Sacch. erythraea* carrying integrative vectors pRT801 (*int*-φBT1) (Gregory et al. 2003), pCLY10 (*int*-VWB) (Bilyk et al. 2016) and pSET152 (*int*-φC31) (Bierman et al. 1992) was isolated and digested with the restriction endonuclease *Nru*I. The enzyme was inactivated by incubation at 65 °C for 10 min, and the fragmented DNA was self-ligated using T4-DNA ligase overnight at 16 °C. The ligase mixture was transformed into *E. coli* GB2005 by electroporation. Plasmids were purified from *E. coli* and verified by endonuclease restriction mapping. Plasmids confirmed to carry the insert of the genomic DNA were sequenced by Sanger using primer pairs pCLY10attSeqF/pCLY10attSeqR, pRT801attSeqF/pRT801attSeqR and pSET152attSeqF/pSET152attSeqR for vectors pCLY10, pRT801 and pSET152, respectively (Table S3).

The WGS reads QC was performed using FastQC, and adapters were removed by SOAPnuke (Chen et al. 2018). The raw data is deposited in NCBI SRA under SRA Experiment SRP673973. Genome sequences were assembled using Newbler v3.0 and annotated with PGAP identified (Silva et al. 2013; Tatusova et al. 2016). Default parameters were used for all software. The assembled data is deposited in GenBank under accession BioProject accession number PRJNA1416820. For WGS-based *attB* identification, the forward and reverse reads were assembled using flash v1.2.11 (Magoc and Salzberg 2011). Both extended fragments and raw reads fastq files were used for subsequent analysis. The raw reads were searched for exact matches (forward and complement) of probe sequences (50 bp) using *grep* (Table S4). Reads matching probe sequences were extracted from the original raw reads fastq file using *awk* (Aho et al. 2024). Sequence matching probe sequence was masked using *sed* command. After masking, remaining sequences were mapped to original reference genome using Geneious Prime v. e2024.0.7 (Dotmatics, USA).

### Construction of pKC1132—191 and pYS191

pWOR191 was purified from *S. lividans* 3215, shotgun sequenced on BGI DNBseq platform with 1 Gb of raw data output (BGI Tech Solutions, Poland), and 10% of the obtained data was used to assemble the sequence of the plasmid. The sequence was annotated using Geneious prime annotation function and pJV1 sequence as a reference (Servin-Gonzalez et al. 1995). The 4.2—kb fragment containing pJV1 replicon was amplified from the pWOR191 by primers pWOR191_Bm_F and pWOR191_Xb_R (Table S3). The PCR product was purified, digested with *Bam*HI and *Xba*I and cloned into the *Bam*HI/*Xba*I-linearized pKC1132 resulting in pKC1132—191. The *Bgl*II/*Xma*I-2.7—kb fragment containing pJV1 replicon was retrieved from the pKC1132—191 and cloned into the *Bgl*II/*Xma*I-linearized pKC1132 to give pYS191 (GenBank accession PX985116). The plasmid copy number was estimated using the genome coverage of the strains carrying corresponding vectors. The raw data was deposited in SRA under SRA Experiment PRJNA1417616.

### Analysis of erythromycin production

For erythromycin production, *Sacch. erythraea* strains were cultivated in 20 ml of liquid TSB medium supplemented with apramycin (50 μg/mL) where required at 30 °C for 3 days on a rotary shaker at 180 rpm. 1.5 mL of pre-culture was seeded into 30 mL of EFM medium in 300-mL flasks and incubated at 30 °C for 7 days. Erythromycin was extracted from the 15 mL of culture with equal volume of ethyl acetate. The organic phase was collected and evaporated on a RV 8 pro V-C Complete (IKA, Germany). The extracts were dissolved in 200 μL of methanol and analysed on an Amazon Speed (Bruker Daltonics, USA) coupled with a Dionex Ultimate 3000 RSLC HPLC (Thermo Scientific, USA). Samples were separated on a BEH C18, 100 × 2.1 mm, 1.7-μm dp column (Waters, Germany). Separation of the 1 μL sample was achieved by a linear gradient of solvent B (acetonitrile + 0.1% formic acid) against solvent A (water + 0.1% formic acid) at a flow rate of 600 μL/min and 45 °C. The gradient started by a 1-min isocratic step at 5% B and then increased to 95% B over 18 min to end up with a 2-min step at 95% B before re-equilibration under the initial conditions. UV spectra were acquired by a DAD detector in the range of 200–600 nm. The mass spectra were acquired in centroid mode ranging from 200 to 2500 m/z at a 2 Hz scan rate. Data was collected and analysed with the Bruker Compass Data Analysis software, version 4.1 (Bruker Daltonics, USA). Three individual clones of each strain were tested. The area of peaks which correspond to different erythromycin derivatives were calculated, and the data was normalised to the wet biomass.

### Plasmids stability evaluation

*Sacch. erythraea* and *S. albidoflavus* strains carrying plasmids pKC1132—191 and pYS191 were grown for three consecutive passages at non-selective conditions on MS agar medium till sporulation. From each passage, the spore suspension was prepared, and the titre of vector-containing cells was determined. For this, cell suspensions with 10^7^–10^8^ cells per mL were plated by serial dilutions on plates with apramycin (50 μg/mL), and the number of apramycin-resistant colonies was counted after 7–9 days of incubation. The experiment was repeated three times, and the stability of the plasmids was assessed as the titre of apramycin-resistance cells in the initial suspension.

## Results

### Conjugation frequency of various vectors into the *Sacch. erythraea* DSM40517

We selected five actinobacterial vectors, three integrative (pCLY10 (*int*-VWB (Bilyk et al. 2016)), pRT801 (*int*-φBT1 (Gregory et al. 2003)), pSET152 (*int*-φC31 (Bierman et al. 1992)) and two replicative (pKC1139 (pSG5 replicon (Muth et al. 1988)), pUWLint31 (pIJ101 replicon (Wehmeier 1995)), to be tested for the efficiency of introduction into *Sacch. erythraea*. All vectors carry the RK2 *oriT* which facilitate the intergeneric conjugation transfer into *Actinobacteria* (Blaesing et al. 2005). As a control, the same vectors were transferred into the *S. albidoflavus* Del14 (Myronovskyi et al. 2018). Prior mating, the titre of recipient cells was established. Conjugation efficiency was calculated as the ratio of obtained transconjugants to a number of recipient cells.

All three integrative vectors were transferred into *Sacch. erythraea* and *S. albidoflavus* strains with different frequencies (Table 1). In the case of *Sacch. erythraea*, vectors based on the VWB and φC31 actinophages demonstrated slightly higher transfer rate than pRT801 (φBT1). The highest frequency of *Sacch. erythraea* transconjugants was observed with pCLY10. At the same time, this plasmid transfer efficiency was significantly lower in case of *S. albidoflavus* Del14 (Table 1).

**Table 1:** Conjugation efficiency of plasmids into *Sacch. erythraea* and *S. albidoflavus*

Plasmid	*Sacch. erythraea* DSM40517	*S. albidoflavus* Del14
pCLY10 (int-VWB)	3.3 ± 0.1 × 10^−6^	7.1 ± 1.7 × 10^−9^
pRT801 (int-φBT1)	1.4 ± 0.9 × 10^−7^	3.5 ± 0.2 × 10^−7^
pSET152 (int-φC31)	9.9 ± 3.5 × 10^−7^	1.2 ± 0.3 × 10^−7^
pKC1139 (pSG5 rep)	NA*	4.0 ± 0.1 × 10^−7^
pUWLint31 (pIJ101 rep)	NA*	5.8 ± 0.3 × 10^−8^

*Not achieved

Despite several attempts (at least 3 for each vector) and protocol modifications, we were not able to obtain *Sacch. erythraea* transconjugants with tested replicative vectors. At the same time, they were introduced into *S. albidoflavus* with a high efficiency. Since both pIJ101 and pSG5 replicons are known to be functional in *Sacch. erythraea*, we assume that the transfer efficiency of pKC1139 and pUWLint31 is significantly lower than that of the integrative ones.

### Cloning and identification of vectors’ integration sites from the genome of *Sacch. erythraea*

The genomic regions flanking the *attB* sites were recovered from chromosomal DNA of *Sacch. erythraea* carrying pCLY10, pRT801 and pSET152 and sequenced. From 20 to 30 independent clones of each plasmid were mapped with restriction endonucleases in order to confirm the capture of genomic region. Surprisingly, the insert was present in 90% of recovered pSET152 (Fig. S1), 80% of pRT801 (Fig. S2) and only 30% of pCLY10 (Fig. S3).

**Fig. 1:**
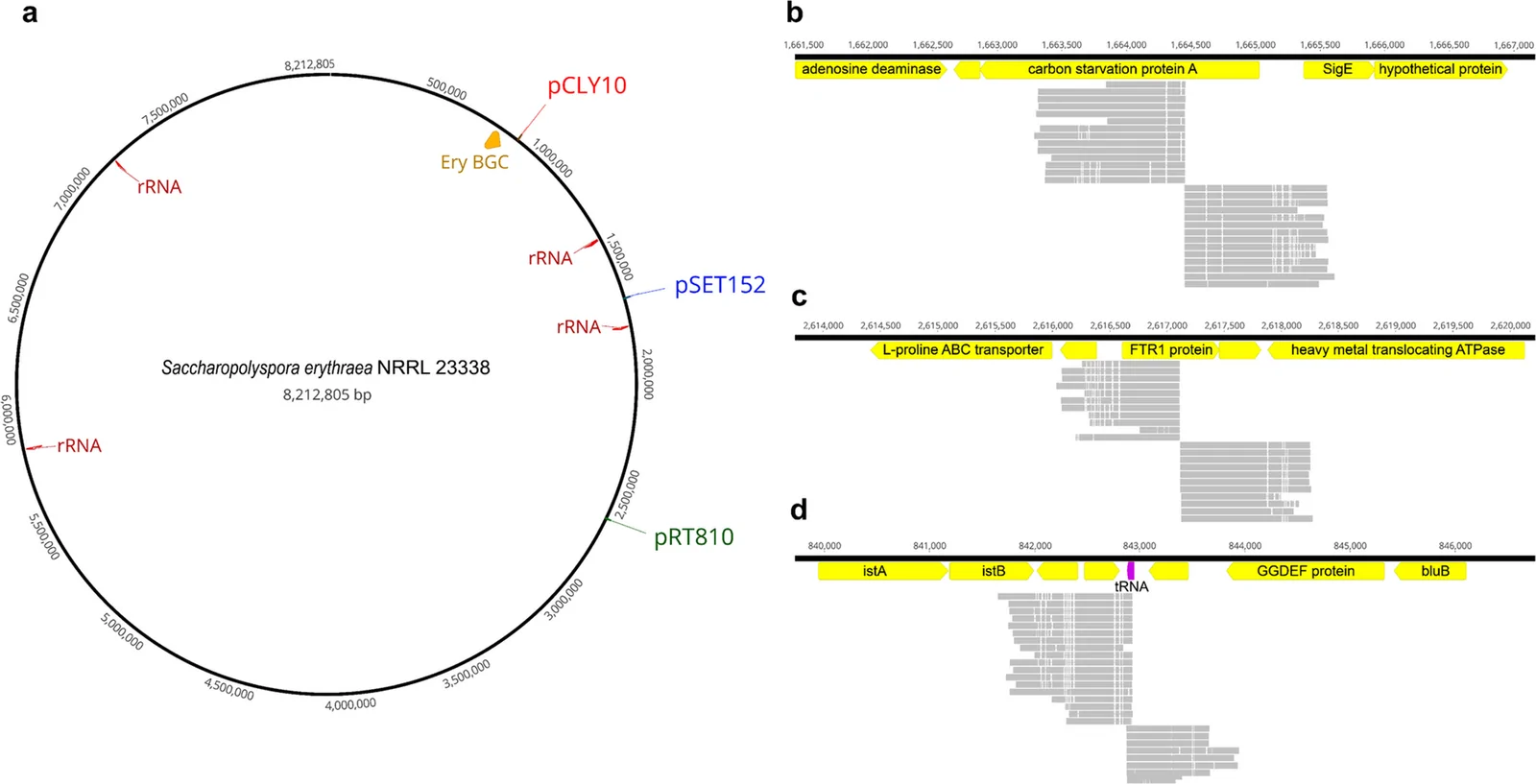
Identification of actinophage-based vectors integration sites in the genome of *Sacch. erythraea*. **a** Schematic circular map of the *Sacch. erythraea* NRRL 23338 genome with indicated locations of pSET152, pRT801 and pCLY10 vectors integration sites. Ery BGC stands for erythromycin biosynthetic gene cluster. Sequences obtained by vector retrieving procedure mapped to the genome of *Sacch. erythraea*: pSET152 within the *cst*A gene (**b**); pRT801 within the gene encoding FTR1 family protein (**c**); and pCLY10 within the tRNA^Arg^ gene (**d**). Grey lines correspond to sequencing reads

**Fig. 2:**
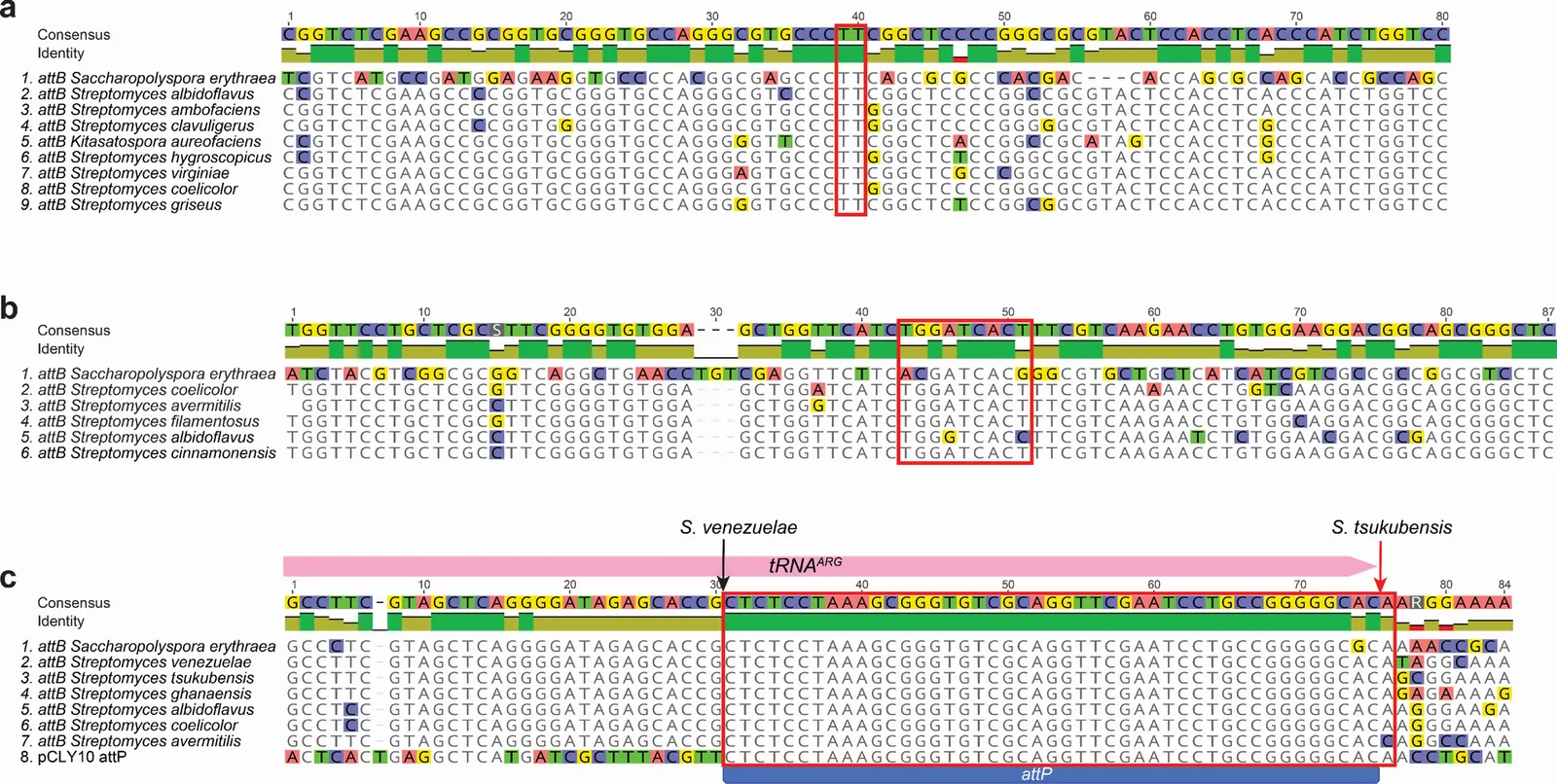
Multiple sequence alignment (MUSCLE) of *attB* sites of studied integrative vectors from *Sacch. erythraea* and different *Streptomyces* species. **a** pSET152 (φC31). **b** pRT801 (φBT1). **c** pCLY10 (VWB). Sequences involved in recombination are highlighted with red frames. In the case of VWB phage, the previously published integration sites are shown with the black (*S. venezuelae*) and red (*S. tsukubensis*) arrows. The sequence common for *attB* and *attP* sites is highlighted with red frame

At least ten clones of each vector were sequenced from primers annealing outward to the *attP* site. Obtained sequences were mapped to the genome of *Sacch. erythraea* (NCBI Reference Sequence: NC_009142.1, Fig. 1a)*.* In the case of pSET152, all sequences from 14 individual clones were mapped to the same region of the genome within the reading frame of the *cst*A gene (locus tag SACE_RS07435, insertion site at 1 664 444 bp) (Fig. 1b). No other regions of the chromosome were retrieved, indicating that in *Sacch. erythraea*, a predominant site of integration of φC31 vectors exists. The alignment of φC31 *attB* sequence from *Sacch. erythraea* and other actinobacteria did not reveal much of a similarity (48–50% of nucleotide identity) (Fig. 2a) (Combes et al. 2002). Thus, the integration of φC31 vectors in the genome of *Sacch. erythraea* occurs at non-canonical (pseudo) *attB* sequence.

In the case of pRT801 11, recovered clones were sequenced and mapped to the genome of *Sacch. erythraea* within the gene encoding the FTR1 family protein (locus tag SACE_RS11775, insertion site at 2 617 156 bp) (Fig. 1c). The integration locus for φBT1 was experimentally confirmed only for *S. coelicolor* (Gregory et al. 2003). The pRT801 vector integrates into the genome of this strain into the SCO4848 gene encoding a putative integral membrane protein. The alignment of φBT1 *attB* sequences from *Sacch. erythraea*,* S. coelicolor* and several other *Streptomyces* species (deduced by homology to SCO4848) showed that *Sacch. erythraea attB* is significantly different (46–48% of identity) and also seems to be a pseudo-*attB* (Fig. 2b).

Eighteen clones of pCLY10 were sequenced and analysed. All of them are mapped within the tRNA^Arg^ gene (locus tag SACE_RS03670) (Fig. 1d). The sequences obtained from forward and reverse primers overlapped by 46 nucleotides. This 46 bp region is present in both *attP* and *attB* sites (Fig. 2c) and is proposed to serve as the recombination site facilitating phage insertion. Unlike the other two actinophage integration systems, the VWB actinophage *attB* is highly conserved among actinobacteria including *Sacch. erythraea* (Fig. 2c).

### WGS-based vector integration sites identification

In order to confirm the identification of integration sites of tested vectors, we have sequenced the genome of *Sacch. erythraea* strains carrying pSET152, pRT801 and pCLY10. One clone of each strain was inoculated into the liquid medium, grown for 3–6 days, and genomic DNA was sequenced. Genomes were assembled to scaffold level. The scaffolds corresponding to the integrative vectors were searched within the obtained data, and in all cases, a single copy of the vector sequence was detected. The *attB* sites identified by the genome analysis in all cases coincide with the loci identified by chromosome-retrieving approach (Fig. S4). However, during the genome assembly, the minor reads are neglected preventing identification of minor sites.

In order to detect the possible minor vector integration sites, the sequences which correspond to the left and right side of *attP* (50 bp) of each vector were used as probes (Table S4). The raw data was screened for the reads containing probe sequences. Identified reads were trimmed to remove the probe and mapped to the genome of *Sacch. erythraea* (Supplementary files 1, 2 and 3)*.* In the case of pRT801 and pCLY10 vectors, all reads mapped to the genome are located in the same sites which correspond to the *attB* loci identified previously (Figs. S5, S6).

**Fig. 3:**
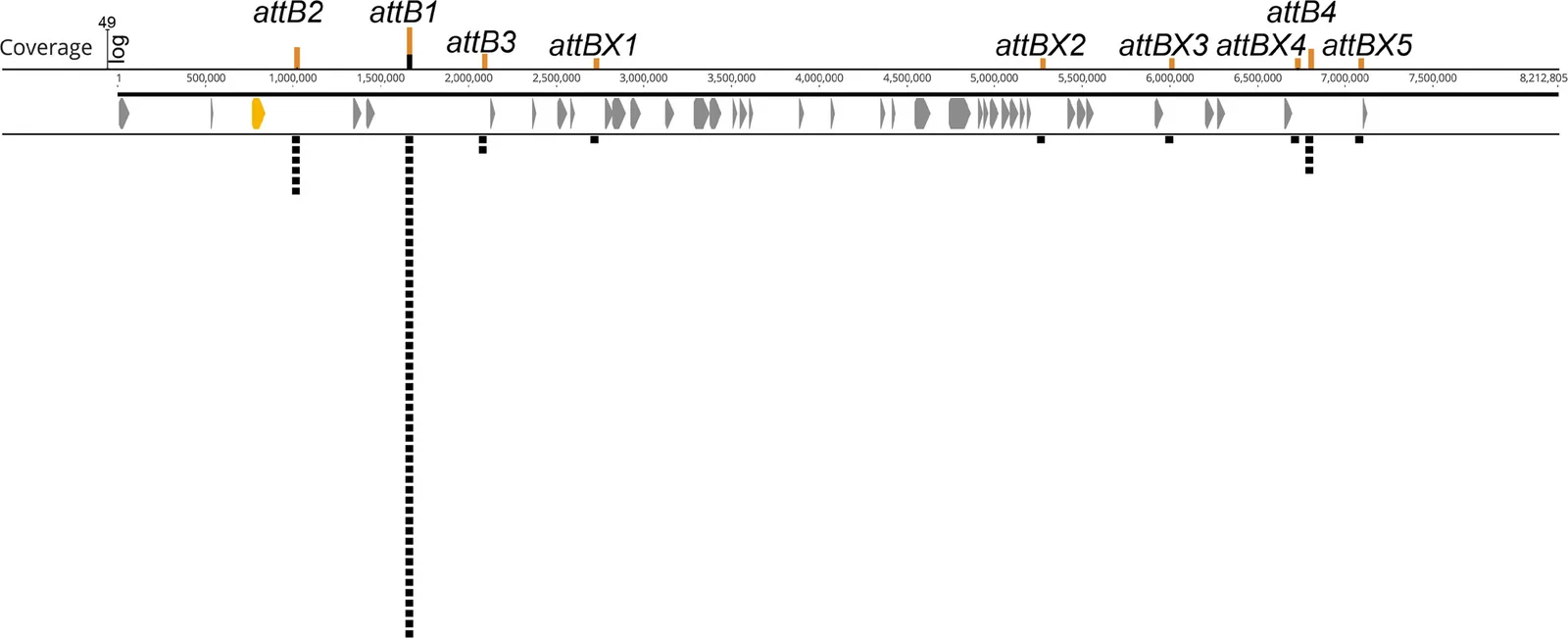
Mapping of reads retrieved with pSET152-attR/L probes to the genome of *Sacch. erythraea.* Each dot represents a single read. Identified pseudo-*attB* loci are highlighted. Grey arrows represent BGCs, orange arrow shows the location of erythromycin BGC

In the case of pSET152, four different loci were identified with at least two reads properly mapped: 53 reads aligned to the region of *cstA* gene which was cloned by the chromosome-retrieving procedure (pseudo-*attB1*); six reads mapped at the end of the putative hypothetical protein encoding gene (locus tag SACE_RS04445, insertion position 1 020 467 bp) (pseudo-*attB2*); two reads were located within the gene coding for putative S8 family serine peptidase (locus tag SACE_RS09365, insertion position 2 089 749 bp) and four reads aligned to the gene coding for putative D-Ala-D-Ala carboxypeptidase family metallohydrolase (locus tag SACE_RS29085, insertion position 6 802 195 bp) (Fig. 3; Fig S7). In addition, five reads were individually mapped to five different regions of the genome (Fig. 3; Fig. S8).

In all cases, a small proportion of selected reads were mapped to the sequence of the corresponding vectors in the regions adjacent to the *attP* site (Table 2). This indicates that all three vectors are excised from the chromosome of the strain and could exist as circular non-replicating DNA molecules. Finally, we have estimated vectors copy number in *Sacch. erythraea*. In the case of pSET152, the plasmid coverage was 152 nucleotides per position, and the genome coverage was 145 nucleotides per position, giving 1.05 copies of the vector per chromosome. The pRT801 is present at 1.18 copies (genome coverage 151 n.t. per position, vector coverage 178 n.t. per position); and pCLY10 showed the highest copy number among the integrative vectors at 1.34 copy per chromosome (genome coverage 150 n.t. per position, vector coverage 202 n.t. per position). In the case of the latter two vectors, these extra copy numbers could be explained by the excision process. However, in the case of pSET152, both excision and multi-copy integration most probably contribute to the observed copy number.

**Table 2:** Mapping of raw reads with *attP* sequences to the genome of *Sacch. erythraea*

Probe name	Total identified reads	Mapped to the genome	Mapped to the vector	Percentage of reads mapped to the vector
pSET152-attR/L	75	70	5	6.6%
pRT801-attR/L	151	138	13	8.6%
pCLY-attR/L	166	153	13	7.8%

### Construction of a new functional replicative vector for *Sacch. erythraea*

The only replicative vector reported to be efficiently maintained by the *Sacch. erythraea* is based on the replicon of pJV1 plasmid from *S. phaeochromogenes* (Yamamoto et al. 1986). However, we were not able to find any pJV1-based shuttle vectors suitable for conjugational transfer into actinobacteria. The *S. lividans* strain carrying the “streptomyces only” pWOR191 plasmid which is direct derivative of pJV1 was kindly provided by Prof. Mark Buttner (JIC StrepStrains (jic.strepstrains@jic.ac.uk, John Innes Centre, Norwich Research Park, Norwich, NR4 7UH, UK) (Bailey et al. 1986). Plasmid was purified and de novo sequenced. After assembly, the plasmid was annotated using pJV1 as a template (GenBank Accession: NC_001759.1, Fig. S9) (Servin-Gonzalez et al. 1995). pWOR191 comprises the entire pJV1 plasmid with a cloned thiostrepton resistance gene for selection in actinobacteria. The plasmid contains the *tra*, *spdB* and *rep* genes required for conjugative transfer and plasmid maintenance.

**Fig. 4:**
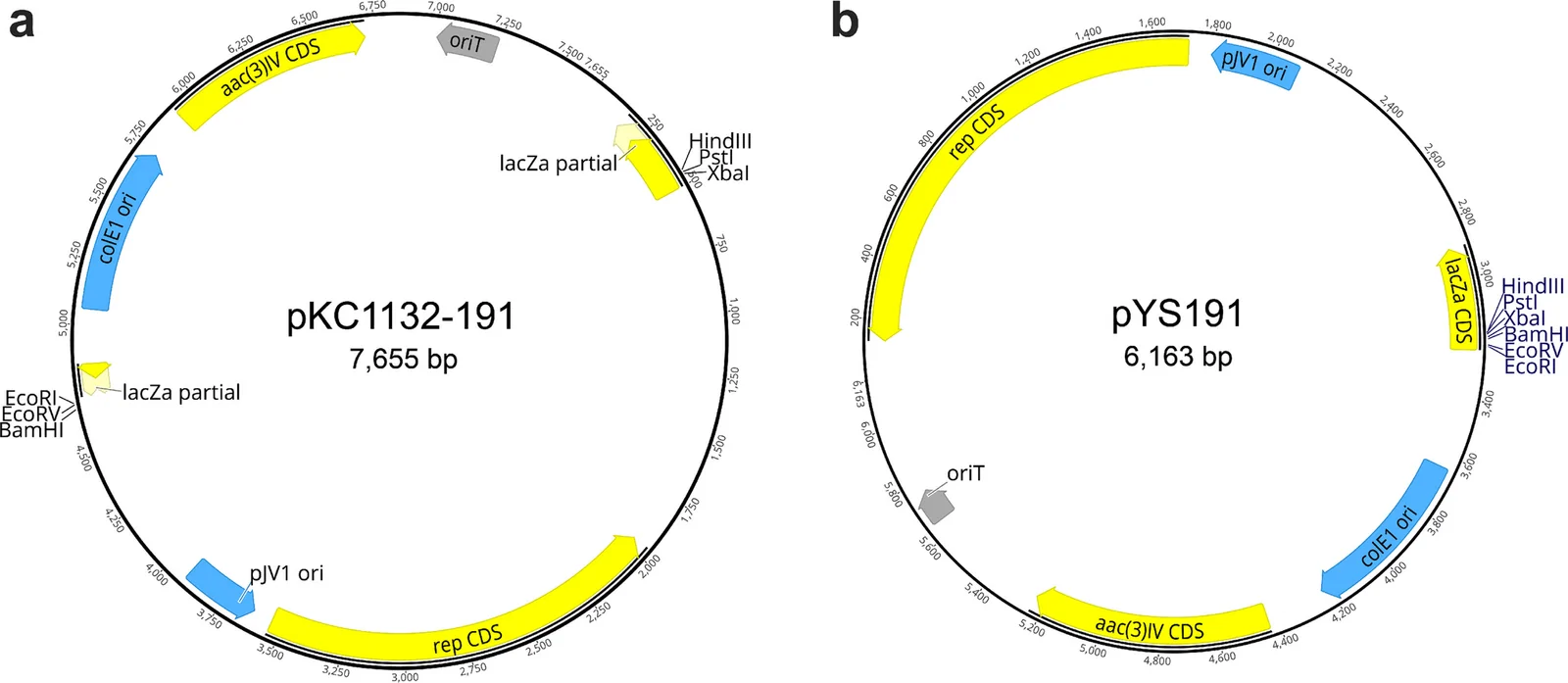
Schematic representation of pKC1132—191 **(a)** and pYS191 **(b)** vectors. Abbreviations: oriT, RP4 (RK2) origin of transfer; *aac(3)IV*, apramycin resistance; pJV1 ori, minimal pJV1 origin of replication; colE1 ori, *E. coli* origin of replication; *lacZa*, *lacZ’* gene with MCS

In order to identify the pJV1 replicon region suitable for vector construction, the 4.2 kb fragment containing oriC and *rep* gene was amplified from pWOR191 and cloned into the multiple cloning site (MCS) of actinobacterial suicide vector pKC1132 (Kieser et al. 2000). Resulting plasmids pKC1132—191 (Fig. 4a) was introduced into *Sacch. erythraea *via conjugation with *E. coli* proving that the cloned fragment contains the replication function. Further analysis of the cloned fragment sequence showed that the nick site (CCTA**GG**TAAA) where the rolling circle replication of the pJV1 is initiated is located just upstream of the *rep* gene making possible to further reduce the size of replicon (Servin-Gonzalez et al. 1995). The 3.4 kbp part of pJV1 replicon was retrieved from pKC1132—191 as a *Xma*I and *Bgl*II fragment and cloned into respective sites of pKC1132. This gave the pYS191 shuttle *E. coli—Streptomyces* replicative vector (6.163 bp) based on minimal pJV1 replicon (*rep*), *E. coli* origin of replication (colE1 ori), carrying apramycin resistance gene (*aac(3)IV*) for selection, *lacZ’* gene (*lacZa*) with convenient MCS and RP4 (RK2) origin of transfer (oriT) (Fig. 4b).

In order to evaluate constructed plasmids performance, pKC1132—191 and pYS191 were transferred via intergeneric conjugation into *Sacch. erythraea* and *S. albidoflavus*. Both vectors were successfully introduced into tested strains (Table 3). However, the transfer efficiency of both vectors was significantly (100-fold) higher in the case of *Sacch. erythraea* than in *S. albidoflavus*. The frequency of *Sacch. erythraea* transconjugants with the constructed replicative plasmids was at the level of integrative vectors pSET152 and pRT801 (Table 1).

**Table 3:** Conjugation frequency of plasmids into *Sacch. erythraea* and *S. albidoflavus* recipients

Plasmid	Recipient strain	
	*Sacch. erythraea* DSM40517	*S. albidoflavus* Del14
pKC1132—191	5.0 ± 0.6 × 10^−7^	1.9 ± 1.0 × 10^−9^
pYS191	9.4 ± 1.4 × 10^−7^	3.2 ± 1.9 × 10^−9^

Finally, we determined the copy number of constructed replicative vector. Strains *Sacch. erythraea* and *S. albidoflavus* Del14 carrying the pYS191 were grown in liquid TSB media for 3 days; total DNA was isolated and subjected to WGS sequencing. The PCR-free library was used to avoid biasing related to sequencing library preparation. Obtained raw data was mapped to the concatenated sequences of the genome of corresponding host strain and pYS191. The plasmid copy number was calculated as a ratio between the read coverage of the chromosome and plasmid. In the case of *Sacch. erythraea*, the plasmid coverage was 377 nucleotides per position, and the genome coverage was 141 nucleotides per position, giving 2.67 copies of the pYS191 per chromosome. In *S. albidoflavus*, the vector median coverage was 1491 nucleotides per position with the average genome coverage of 202 nucleotides per position, resulting in 7.38 to 1 ratio. This data suggests that pYS191 is maintained in the *Sacch. erythraea* DSM40517 at 2–3 copies per chromosome and in *S. albidoflavus* at 7–8 copies per chromosome.

The pYS191 vector was tested for its ability to be used in several other species of actinobacteria. It was successfully introduced into a number of strains including *Streptomyces lividans* TK24 (conjugation frequency – 4.3 ± 1.0 × 10^−9^), *Couchioplanes caeruleus* DSM43634 (5.6 ± 1.5 × 10^−9^), *Streptomyces filamentosus* NRRL 15998 (4.6 ± 1.7 × 10^−8^) and *Saccharothrix australiensis* DSM43800 (2.6 ± 2.5 × 10^−10^). However, we did not succeed to obtain the pYS191 transconjugants with *Nonomuraea polychrome* NRRL B-16243, *Nocardia tenerifensis* DSM44704, *Streptomyces eurocidicus* DSM40604, *Streptomyces lavendofoliae* NRRL B-3371, *Streptomyces canus* DSM40017 and *Streptomyces griseoplanus* DSM40009.

### Vectors stability and influence on erythromycin biosynthesis

**Fig. 5:**
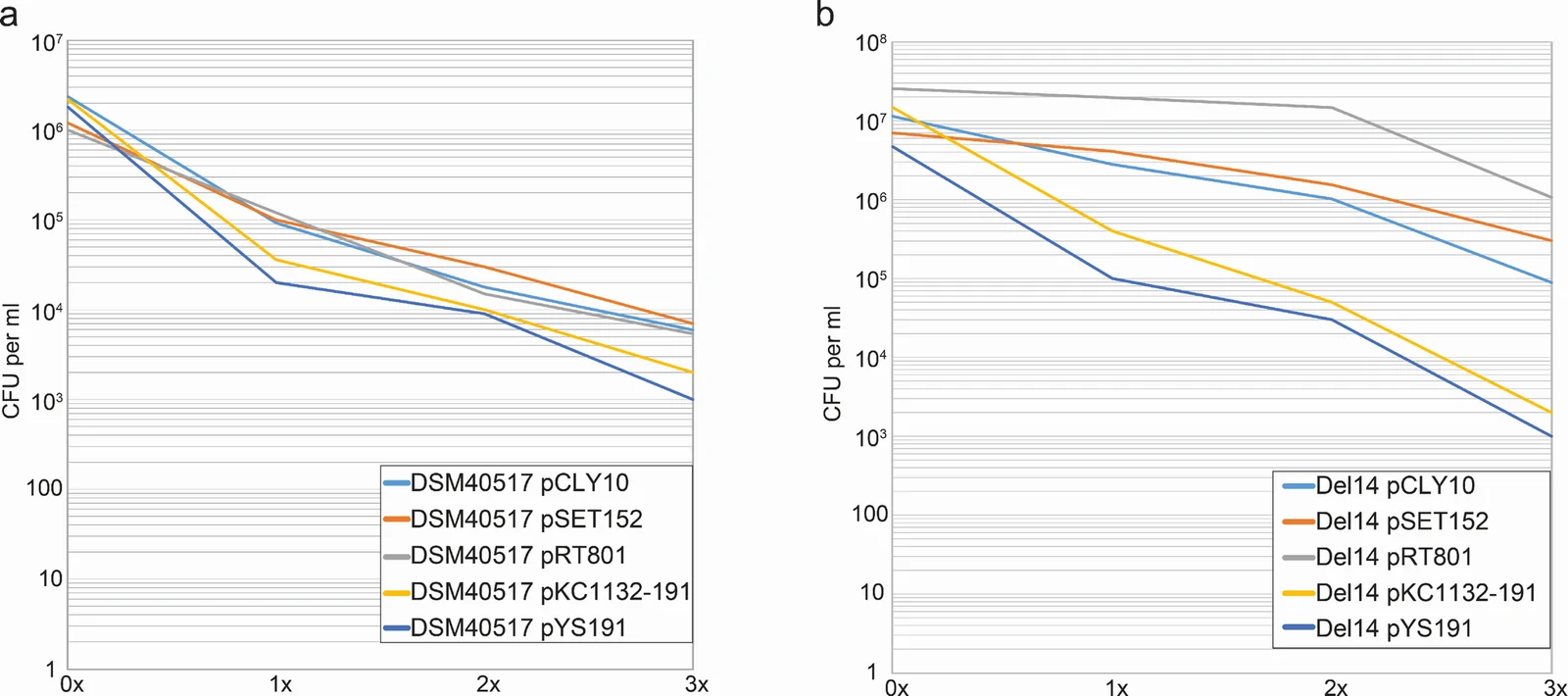
Dynamics of replicative and integrative vectors loss under non-selective conditions in *Sacch. erythraea* DSM40517 (**a**) and *S. albidoflavus* Del14 (**b**)

To assess the performance of pKC1132—191 and pYS191, we evaluated their inheritance in *Sacch. erythraea* DSM40517 and *S. albidoflavus* Del14 in the absence of selective pressure, as well as their impact on erythromycin production. Strains of *Sacch. erythraea* and *S. albidoflavus* carrying aforementioned vectors were subjected to four sequential cultivations on antibiotic-free solid medium. After each passage, from 10^7^ to 10^8^ spores were plated on media supplemented with apramycin, and the titre of.

plasmid carrying cells was calculated. The experiment was performed in triplicate. The pKC1132—191 and pYS191 exhibited similar loss dynamics in both strains (Fig. 5). After first passage under non-selective conditions, the titre of vector-carrying cells decreased 60–90-fold in the case of *Sacch. erythraea* and 35–45-fold in the case of *S. albidoflavus.* After three passages, only 0.05–0.09% of *Sacch. erythraea* and 0.01–0.02% of *S. albidoflavus* cells retained the vectors. Such high loss frequency is beneficial when the transient expression of the gene of interests is needed. It allows elimination of the vectors in the absence of selective pressure, while the presence of selective antibiotic in the medium ensures their stable maintenance. At the same time, we did not observe the drop in apramycin-resistant cell titre when the strains were cultured under the selective pressure indicating that both vectors are stably maintained in the presence of antibiotic (data not shown).

The loss of integrative vectors was also observed in both species (Fig. 5). However, the trends were different in case of *S. albidoflavus* and *Sacch. erythraea*. When in S*. albidoflavus*, the titre of apramycin-resistant cells carrying pCLY10 and pSET152 dropped to 4–7% after three passages; in the case of *Sacch. erythraea*, the vectors loss was more significant ending at 0.2–0.5% of vector carrying cells from initial cell count. The VWB integration system based pCLY10 demonstrated a slightly lower vector loss rate when comparing with the two other integrative vectors in *Sacch. erythraea*.

**Fig. 6:**
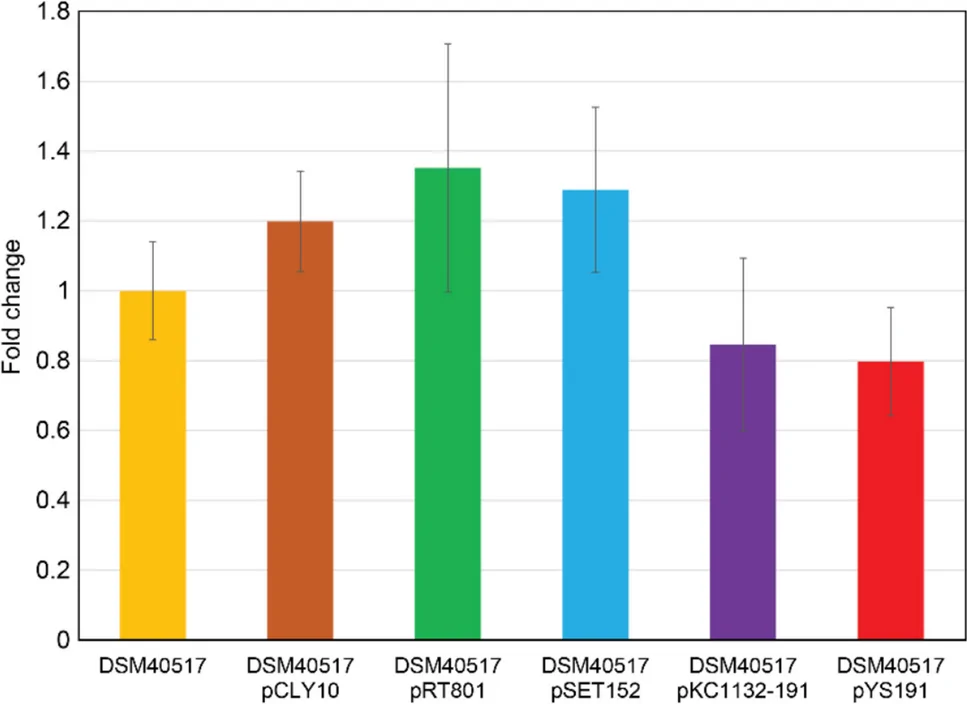
Effect of integrative and replicative vectors on erythromycin production levels in *Sacch. erythraea* strain

Given that both integrative and replicative vectors are known to influence secondary metabolites production, the effect of studied vectors on erythromycin production by *Sacch. erythraea* was tested. Three independent clones of strains carrying each vector were grown in production medium, and erythromycin was extracted after 7 days of cultivation. Extracts were analysed using high-resolution liquid chromatography-mass spectrometry (LC–MS). Erythromycin production was quantified as a sum of area of the peaks which correspond to erythromycin A (RT 6.8 min, m/z = 734.46), erythromycin B (RT 7.6 min, m/z = 718.46) and anhydroerythromycin A (RT 8.1 min, m/z = 716.45) (Fig. S10). The data was normalised to the weight of wet biomass. The total erythromycins yield of parental *Sacch. erythraea* DSM40517 strain was taken as 100%. The levels of erythromycin production by all studied strains are presented in Fig. 6. In all cases, neither integrative nor replicative vectors affected erythromycin accumulation. The observed differences were small and not statistically significant.

## Discussion

*Sacch. erythraea* is one of the model species of actinobacteria and has been used for decades as a platform for the production of the highly valuable antibiotic erythromycin and for studying the properties, structure, and functioning of modular polyketide synthases (Sayed et al. 2020). The detailed investigation of erythromycin biosynthesis resulted in our current understanding of the mechanistic and biochemical aspects of functionality of modular enzymes involved in biosynthesis of bacterial natural products (Leadlay et al. 1993). Despite that, there is an obvious lack of knowledge about vectors behaviour and functional properties in this species*.* In this work, we have systematically investigated the properties of different integrative vectors and constructed the new replicative vector to be used for gene cloning in *Sacch. erythraea*.

The actinophage-based integrative vectors are currently the most popular tool for gene delivery into many actinobacteria strains. Different vectors with different properties and capacity (cosmids, BACs, etc.) exist which employ the site-specific recombination-based genome integration (Rebets et al. 2017). These vectors are considered to have a higher cloning capacity (primarily due to low copy number) and to be stably maintained due to insertion into the chromosome. The integration system of the *Streptomyces* temperate phage φC31 is by far the most used in integrative vectors with the pSET152 being one of the most popular among them (Bierman et al. 1992). pSET152 is the first-choice vector for gene cloning in *Sacch. erythraea* and other *Saccharopolyspora* species (Wang et al. 2008). At the same time, the limited use of actinophage-based vectors in *Sacch. erythraea*, especially VWB and φBT1, is at least partially due to the widespread belief that the canonical *attB* sites are absent in the genome of the strain. For instance, the earlier report has stated that pSET152 vector integrates into up to seven different loci in *Sacch. erythraea* due to the lack of *attB* site (Rodriguez et al. 2003). Our data, support this observation. The φC31 *attB* in *Streptomyces* genomes is located within the ORF gene coding for the chromosome condensation protein (SCO3798 in case of *S. coelicolor*) (Combes et al. 2002). It is also known that the φC31 integrase can recognise somewhat different sequences since in many cases in the absence of the main *attB* site, the phage and φC31-based vectors integrate into so-called pseudo-*attB* (Bilyk and Luzhetskyy 2014; Combes et al. 2002). Indeed, *Sacch. erythraea* lacks the typical φC31 *attB* sequence, and pSET152 is inserted into at least four different loci with the different efficiency. Surprisingly, there is little to no similarity between these four sequences. Most often (76% cases), the vector is inserted into the pseudo-*attB1* site which has 48–50% similarity to the canonical *Streptomyces* sequence (Figs. 1 and 2). It contains the highly conserved CCC(TT) sequence participating in recombination process. This site is located within the *cstA* gene encoding carbon starvation-inducible (CstA) protein. It is involved in stringent response in other bacteria; however, it is poorly studied in actinobacteria (Schultz and Matin 1991). The insertion of pSET152 is leading to gene disruption; however, it has no visible effect on *Sacch. erythraea* growth or erythromycin production (Fig. 6). Three other pseudo-*attB* sites are less efficient in facilitating the recombination with the *attP*, when compared to the pseudo-*attB1.* However, in some cases, two or more pseudo-*attB* sites can be occupied simultaneously, resulting in the insertion of multiple copies of the vector into the strain chromosome (Bilyk and Luzhetskyy 2014; Combes et al. 2002). In addition, we identified five more loci that could potentially serve as φC31 *attB* sites in the *Sacch. erythraea* genome. However, the frequency of vector insertion into these sites is extremely low.

The other two integrative systems are rarely used in *Sacch. erythraea.* In the case of φBT1-based vectors, the low efficiency of transfer originally observed led to the construction of the dedicated strain by inserting the *Streptomyces*-like *attB* sequence into the chromosome of the strain (Lu et al. 2020). In *S. coelicolor*, the φBT1 *attB* is located within the SCO4848 gene encoding a putative integral membrane protein (Gregory et al. 2003). The φBT1 integration was generally accepted to be neutral to the strain phenotype; however, the later report has shown that vector is causing delay in sporulation of the strain due to the polar effect on downstream gene SCO4849 (Gonzalez-Quinonez et al. 2016). In the case of *Sacch. erythraea*, the pRT801 vector integrates into a single locus located within the SACE_RS11775 gene. We achieved a relatively high rate of plasmid transfer into the strain, which clearly demonstrates the feasibility of using this system for gene cloning in *Sacch. erythraea*. The φBT1 *attB* also has a low degree of similarity with the sequences from *Streptomyces* species (45–49%) thus placing it into the pseudo-*attB* group.

The VWB actinophage attachment site is located within the tRNA^Arg^ gene in the genomes of *S. venezuelae*, *S. ghanaensis* and *S. tsukubaensis* (Chen et al. 2012; Ostash et al. 2009; Van Mellaert et al. 1998). In the case of *Sacch. erythraea*, pCLY10 was found to integrate within the same tRNA^Arg^. However, the exact location of the integration event is difficult to determine from existing data. In the case of *S. tsukubaensis*, it was suggested to be one nucleotide downstream of the gene (Chen et al. 2012). However, in this study, the integration region was identified by PCR amplification of *attL* region, which might cause the misinterpretation of results. We used the direct cloning of the *attB* site from *Sacch. erythraea* which allows sequencing both *attL* and *attR* regions. Mapping of obtained sequences to the genome resulted in 46 bp overlap between *attL* and *attR* raw reads (Fig. 2c). This repeat arises from the *attP* sequence which is 100% identical to the part of the tRNA^Arg^ gene (Fig. 1d). We think that the vector integration occurs within this 46 bp sequence splitting the tRNA gene into two parts with *attR* carrying the fully functional copy of the tRNA^Arg^ gene. The fact that *Sacch. erythraea* contains the canonical VWB *attB* site may be the reason for the observed higher conjugation frequency of pCLY10 compared to the other two integrative vectors.

During cloning of vectors’ integration sites, we observed a large number of plasmids recovered from *Sacch. erythraea* lacking the chromosome insert. This leads us to think that integrative vectors may exist in *Sacch. erythraea* cells in a free non-replicating form resulting from the excision activity of the corresponding integrases. It is generally accepted that the integrative vector insertion in actinobacteria is unidirectional (Pokhilko et al. 2016). In the case of φC31 integrase, an additional phage protein gp3 which serves as a recombination directionality factor (RDF) is required for reverse recombination between *attL* and *attR* sites leading to phage/vector excision (Khaleel et al. 2011). However, recently, the φC31 integrase was shown to act as an excisionase in *S. lividans* even without additional factors resulting in approximately 5% of cells lacking pSET152 vector (Duan et al. 2025). In fact, we found that approximately 6–7% of pSET152 exists in a free, non-replicating state in *Sacch. erythraea* culture (Table 2). Although, the φBT1 integrase was shown to have a low excisionase activity in vitro (Zhang et al. 2008)*,* we found that 8–9% of the vector is in a free extrachromosomal form. Both φC31 and φBT1 integrases belong to the large serine recombinases family (Khaleel et al. 2011; Zhang et al. 2008). The VWB actinophage integrase differs from φC31 and φBT1 and belongs to tyrosine recombinases family. These enzymes often but not always require an additional excisionase (xis) protein in order to conduct the phage excision (Jayaram et al. 2015). However, no gene encoding such protein was identified within the VWB genome (Van Mellaert et al. 1998). The FLP and CRE enzymes belong to the same family of recombinases and perform site-specific excision without employing any co-factors. In fact, we observed that a significant proportion (7–8%) of VWB-based pCLY10 vector in *Sacch. erythraea* also exists as an extrachromosomal form suggesting the high frequency of vector excision. Furthermore, all three integrative vectors are lost from the *Sacch. erythraea* and *S. albidoflavus* when grown without antibiotic selection (Fig. 5). Our data indicate the need for antibiotic selective pressure even for integrative vectors to prevent their loss. The two-component vector systems facilitating the removal of integrase gene after recombinant strain construction could be considered as alternative when a stable expression construct integration is desired (Duan et al. 2025; Herrmann et al. 2012).

The replicative vectors are still indispensable when the transient gene expression is needed for genome engineering. This is especially true when it comes to the difficult-to-manipulate actinobacteria (Gomez-Escribano et al. 2025). We have constructed the new replicative shuttle vector pYS191 which could be efficiently transferred into many actinobacterial strains via intergeneric conjugation. The vector is based on the pJV1 replicon known to be the stably maintained in *Sacch. erythraea* under selective pressure (Yamamoto et al. 1986). Although, the pJV1 replicon was described as a high-copy number (about 150 per chromosome (Bailey et al. 1986)), our data shows that the pYS191 is present in *Sacch. erythraea* and *S. albidoflavus* at low copy number (2–3 and copies 7–8 copies per chromosome, respectively)*.* The pYS191 has low or no effect on erythromycin production in *Sacch. erythraea* and is quickly lost when the strain is cultivated under non-selective conditions. After three passages in the absence of antibiotic, 99.95% of cells lose the plasmid. This makes pYS191 a useful tool for transient gene expression in *Sacch. erythraea* and other actinobacteria, expanding the repertoire of tools for genetic engineering of these important microorganisms.

## Supplementary Information

Below is the link to the electronic supplementary material.

ESM 1 (FASTA 35.2 KB)

ESM 2 (FASTA 31.7 KB)

ESM 3 (FASTA 16.4 KB)

ESM 4 (PDF 1.38 MB)
