# Supplementary material for: Evaluation of vector system in Saccharopolyspora erythraea and construction of new replicative vector
Source: Appl Microbiol Biotechnol. 2026 May 14;110:208. doi: 10.1007/s00253-026-13858-2 (PMC13346255; doi:10.1007/s00253-026-13858-2)
Supplement: Supplementary file 1 [file 253_2026_13858_MOESM4_ESM.pdf]

Supporting Information

Evaluation of vector system in *Saccharopolyspora erythraea* and construction of new replicative vector

Yana Nohach[1,2], Markiyan Samborskyy[1,3], Anja Palusczak[4], Andriy Luzhetskyy[1,4], Victor Fedorenko[2], Yuriy Rebets[1,2,3]

[1] German-Ukrainian Core of Excellence in Natural Products Research (CENtR), Zelena Street 20, 79005, Lviv, Ukraine

[2] Department of Genetics and Biotechnology, Ivan Franko National University of Lviv, Hrushevsky Street 4, 79005 Lviv, Ukraine

[3] Explogen LLC, Volodymyra Velykogo street 16, 79053 Lviv, Ukraine

[4] Pharmazeutische Biotechnologie, Universität des Saarlandes, Campus, Geb. C2.3, 66123 Saarbrücken, Germany

Table S1 List of strains used in this study

**Strains**	**Characteristics**	**Reference or source**
Sacch. erythraea DSM40517	Wild type, producer of erythromycin	DSMZ, Germany
S. albidoflavus Del14	Strain with deletion of 14 native BCGs	(Myronovskyi et al., 2018)
S. lividans 3215	Strain carrying plasmid pWOR191	John Innes Centre, UK
E. coli WM6026	*lacIq rrn*B3 Δ*lac*Z4787 *hsd*R514 Δ*ara*BAD567 Δ*rha*BAD568 *rph*-1 *att*λ::pAE12(Δ*ori*R6K-*cat*::Frt5), Δ*end*::Frt *uid*A(ΔMlul)::pir *att*HK::pJK1006(Δ*ori*R6K-*cat*::Frt5; *trf*A::Frt) Δ*dap*A::Frt	(Lal et al., 2019)
E. coli GB2005	F-*mcr*A Δ(*mrr*-*hsd*RMS-*mcr*BC) *φ*80*lac*ZΔM15 Δ*lac*X74 *rec*A1 *end*A1 *ara*D139 Δ(*ara, leu*)7697 *gal*U *gal*K λ *rpsLnup*G*fhu*A::IS2 *rec*ET *redα*, phage T1-resistent	(Tu et al., 2016)

Table S2 List of plasmids used in this study

Plasmids	Description	Source
pCLY10	Integrative vector, derivative of the pCLY9, Am^{R}, *ori*15A, int^{VWB}, *att*P, RP4*ori*T, LEU2, CEN6-ARS4 (Bilyk, Sekurova, Zotchev, & Luzhetskyy, 2016)	Prof. Sergey Zotchev, University of Vienna, Austria
pRT801	Integrative vector, Am^{R}, *ori*T, $int^{\varphi BT1}$	(Gregory, Till, & Smith, 2003)
pSET152	Integrative vector, Am^{R}, *lac*Zα MCS, rep^{pUC}, *ori*T, $int^{\varphi C31}$	(Bierman et al., 1992)
pUWLint31	Replicative vector, Amp^{R}, Tsr^{R}, pUWLCREdeltaKpnI with the *Xba*I/*Bam*HI fragment of pKHint31 containing *int*	(Myronovskyi, Rosenkranzer, & Luzhetskyy, 2014)
pKC1139	Replicative vector, Am^{R}, *lac*Zα MCS, rep^{pSG5}, *ori*T, rep^{pUC}, *gus*A	(Myronovskyi, Welle, Fedorenko, & Luzhetskyy, 2011)
pKC1132	Non-replicative vector, derivative of pOJ260 with deletion between the *Kpn*I to *Spe*I sites, Am^{R}, *lac*Zα MCS, RK2*ori*T (Bierman et al., 1992)	HIPS, Germany
pWOR191	Derivative of pJV1, contains the *rep*, *tra*B, *tra*A, *tra*R, *spd*B1, *spd*B2 and *spd*B3 genes, Tsr^{R}	John Innes Centre, UK
pKC1132-191	Derivative of pKC1132 with replicon (*rep*) from pWOR191, cloned between *Xba*I and *Bam*HI sites within the *lac*Z sequence	This work
pYS191	Derivative of pKC1132 with replicon (*rep*) from pKC1132/191Rep, cloned between *Xma*I and *Bgl*II sites	This work

Table S3 List of primers used in this study

Primers	Primer sequence 5´-3´	Used for:
pCLY10attSeqFnew	ACATCACTCTTTCGAGCGAC	*attB* sequencing
pCLY10attSeqRnew	CTCACCGAGACTCACTGAAA	*attB* sequencing
pRT801attSeqF	AGCGGAAAAGATCCGTCGAC	*attB* sequencing
pRT801attSeqR	GATGAAGGGCGACATGCCTA	*attB* sequencing
pSET152attSeqF	TACTGACGGACACACCGAAG	*attB* sequencing
pSET152attSeqR	CACAACCCCTTGTGTCATGT	*attB* sequencing
pWOR191_Bm_F	ATGGATCCTCGCACAGTTCCTGGACTTT	pJV1 replicon cloning
pWOR191_Xb_R_new	ATTCTAGAAGGAGCACGGTCACCAGAAT	pJV1 replicon cloning
191Rep_chk_F_new	TTTGCGGAAGTGGCAGTCGT	pJV1 replicon cloning
191Rep_chk_R_new	AACTGGTACCAGTTACGCCC	pJV1 replicon cloning

Restriction sites are underlined

Table S4 Probe sequences used for *attB* sites identification

Vector	Sequence	Name
pSET152	CACGTTTTCCCAGGTCAGAAGCGGTTTTCGGGAGTAGTGCCCCAACTGGG	*attP* left
pSET152	CCGGTCACAACCCCTTGTGTCATGTCGGCGACCCTACGCCCCCAACTGAG	*attP* right
pRT801	GCTGGCGCCGGACGGGGCTTCAGACGTTTCGGGTGCTGGGTTGTTGTCTCT	*attP* left
pRT801	ATGGGCGCTGACCTGCGCTTTCTTTTGGGAACATTGGTGGTGCTGAGTAG	*attP* right
pCLY10	GAACGGGGGGCCGTTGACTCCCTGACCTGCGATGCAGGTTGTGCCCCCGG	*attP* left
pCLY10	ACTCACTCAGACTCACTGAGGCTCATGATCGCTTTACGTTCTCTCCTAAA	*attP* right

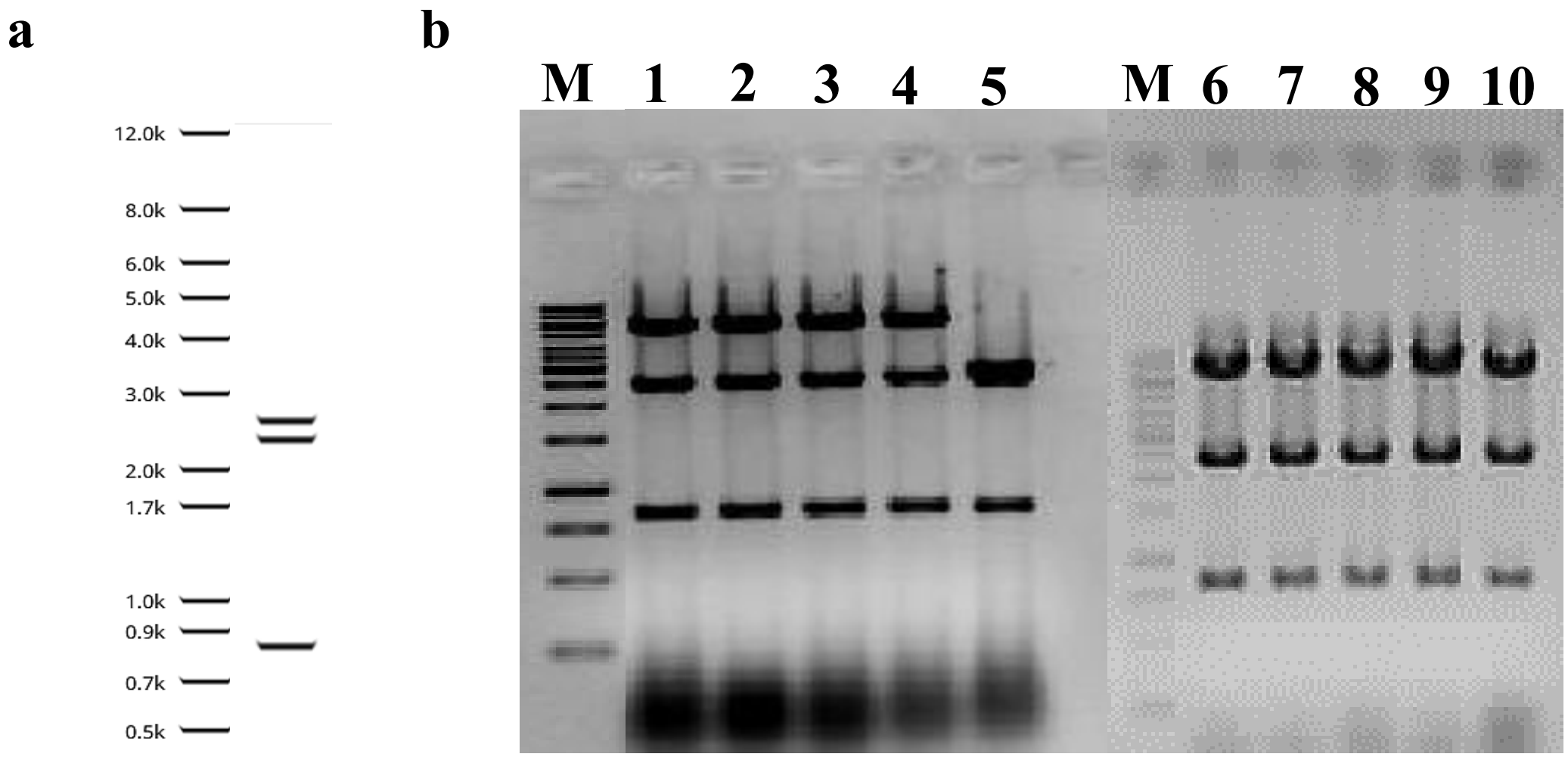

Fig. S1 Verification of pSET152 vector by restriction analysis with *Pst*I. Digestion of the native vector produced fragments of 2.6kbp, 2.3kbp and 0.8kbp, whereas plasmids with chromosomal inserts showed larger or additional fragments. **a.** Visualization of predicted fragments after digestion of pSET152 by *Pst*I with Ladder 1kb Plus (NEB) made by Geneious Prime v. e2024.0.7. **b.** pSET152 without chromosomal insert (lane 5), pSET175 with chromosomal insert (lanes 1-4, 6-10), M – Ladder 1kb (Thermo Fisher Scientific, USA)

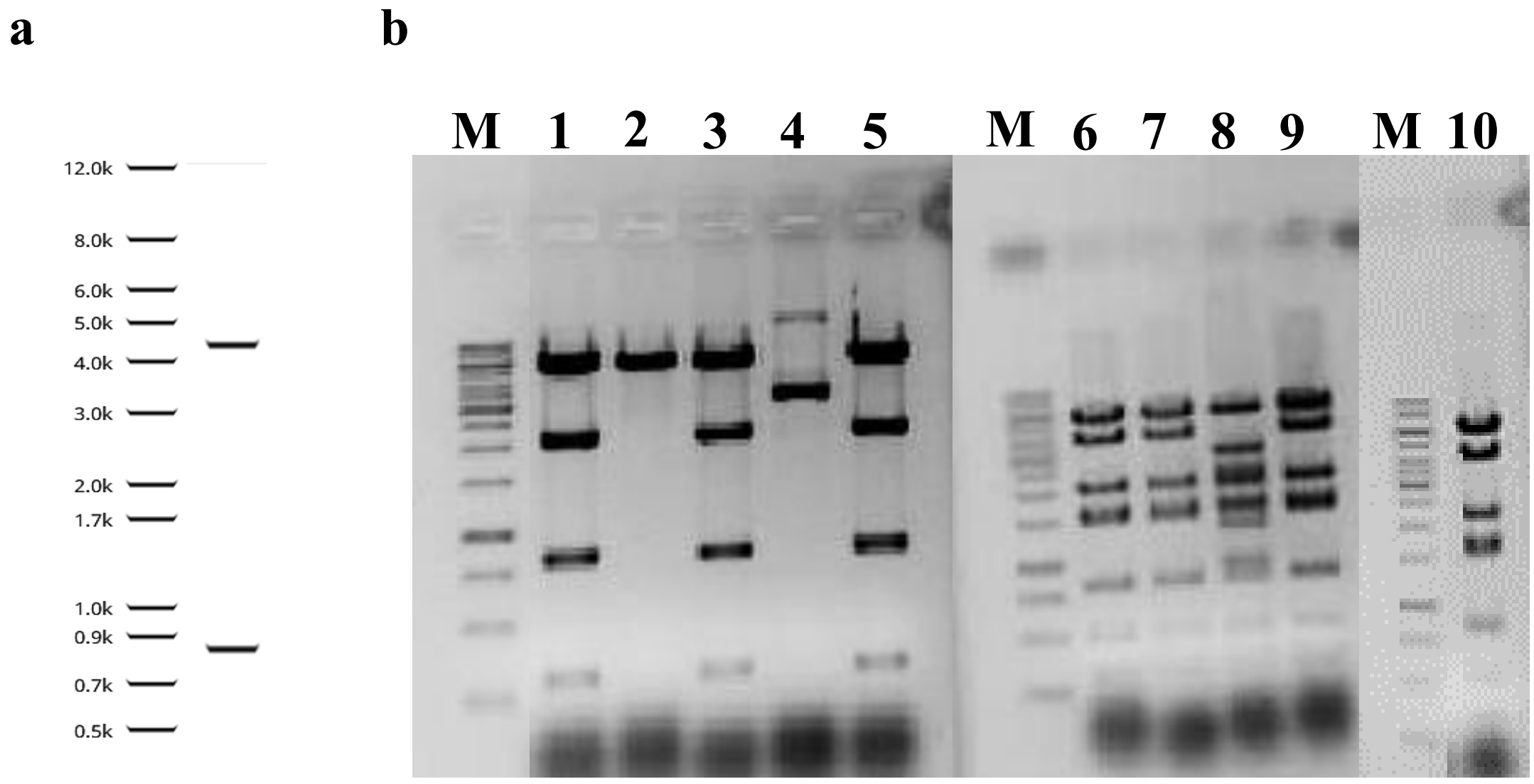

Fig. S2 Verification of pRT801 vector by restriction analysis with *Pst*I. Digestion of the native vector produced fragments of 4.3kbp and 0.8kbp, whereas plasmids with chromosomal inserts showed larger or additional fragments. **a.** Visualization of predicted fragments after digestion of pRT801 by *Pst*I with Ladder 1kb Plus (NEB) made by Geneious Prime v. e2024.0.7. **b.** pRT801 without chromosomal insert (lanes 2, 4), pRT801 with chromosomal insert (lanes 1, 3, 5, 6-10), M – Ladder 1kb (Thermo Fisher Scientific, USA)

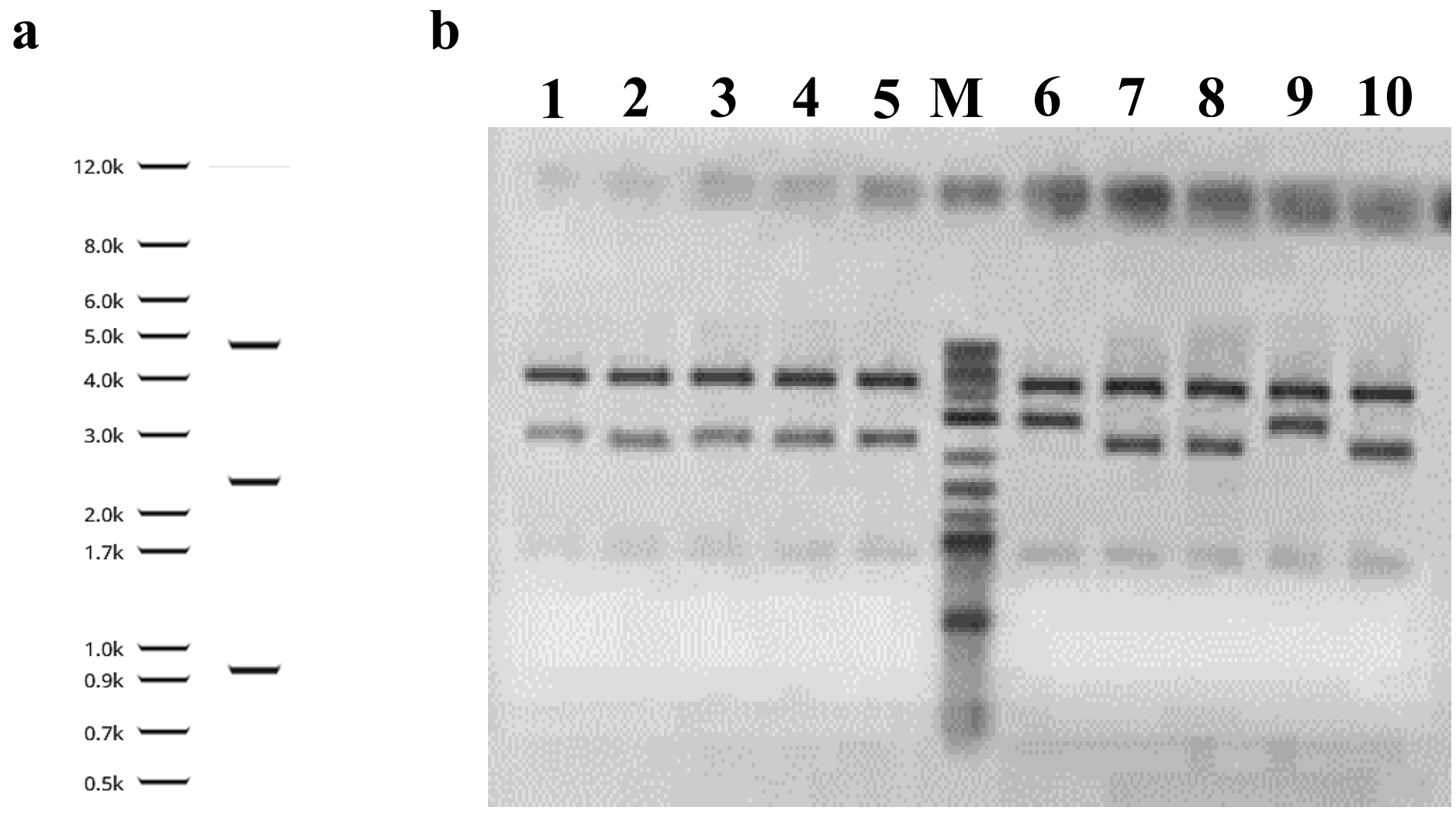

Fig. S3 Verification of pCLY10 vector by restriction analysis with *Xba*I. Digestion of the native vector produced fragments of 4.7kbp, 2.3kbp and 0.9kbp, whereas plasmids with chromosomal inserts showed larger or additional fragments. **a.** Visualization of predicted fragments after digestion of pCLY10 by *Xba*I with Ladder 1kb Plus (NEB) made by Geneious Prime v. e2024.0.7. **b.** pCLY10 without chromosomal insert (lanes 1-5, 7, 8, 10), pCLY10 with chromosomal insert (lanes 6, 9), M – Ladder 1kb (Thermo Fisher Scientific, USA)

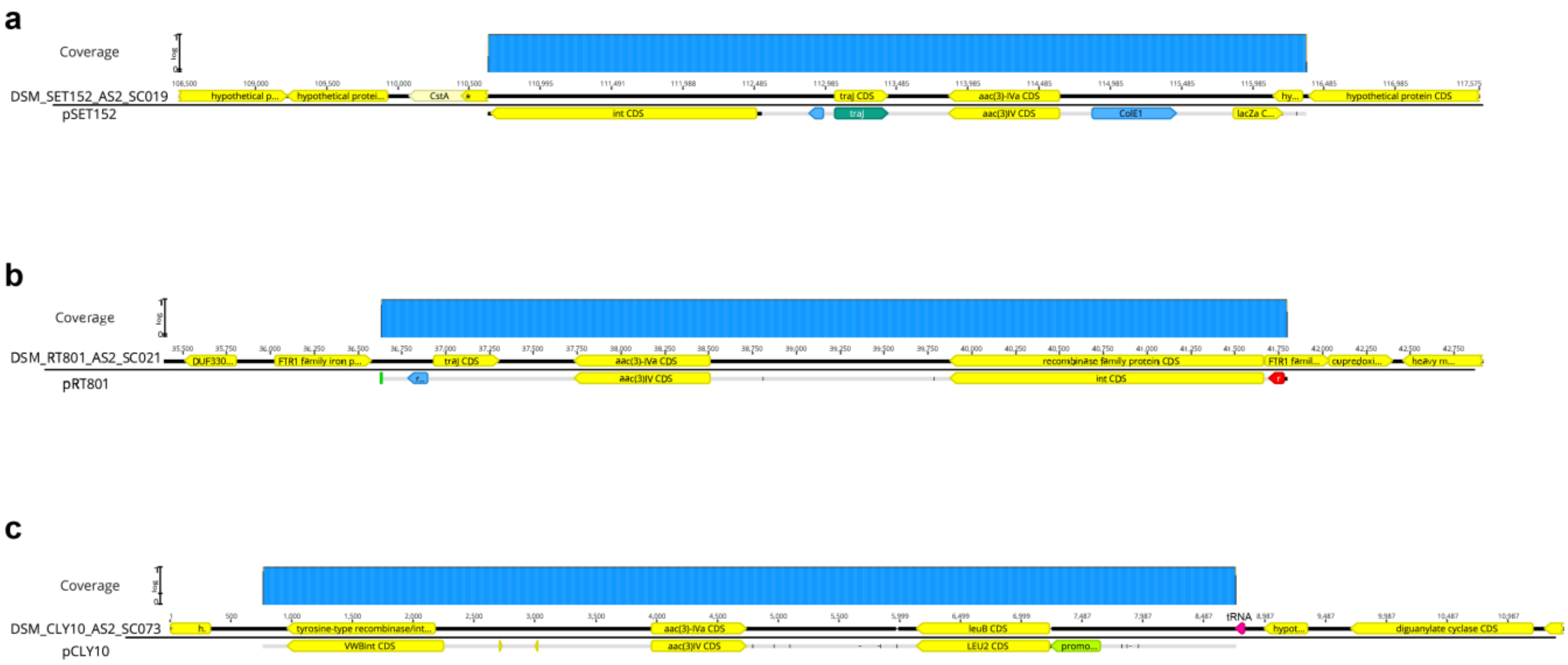

Fig. S4 Mapping of vectors' sequences to the assembled genomes of *Sacch. erythraea* strains carrying corresponding vectors integrated into the chromosome. **a.** *Sacch. erythraea* pSET152 contig SC019 with mapped pSET152. **b.** *Sacch. erythraea* pRT801 contig SC021 with mapped pRT810. **c.** *Sacch. erythraea* pCLY10 contig SC073 with mapped pCLY10. In all three cases the integration sites' location coincides with the *attB* loci identified by chromosomal retrieving approach

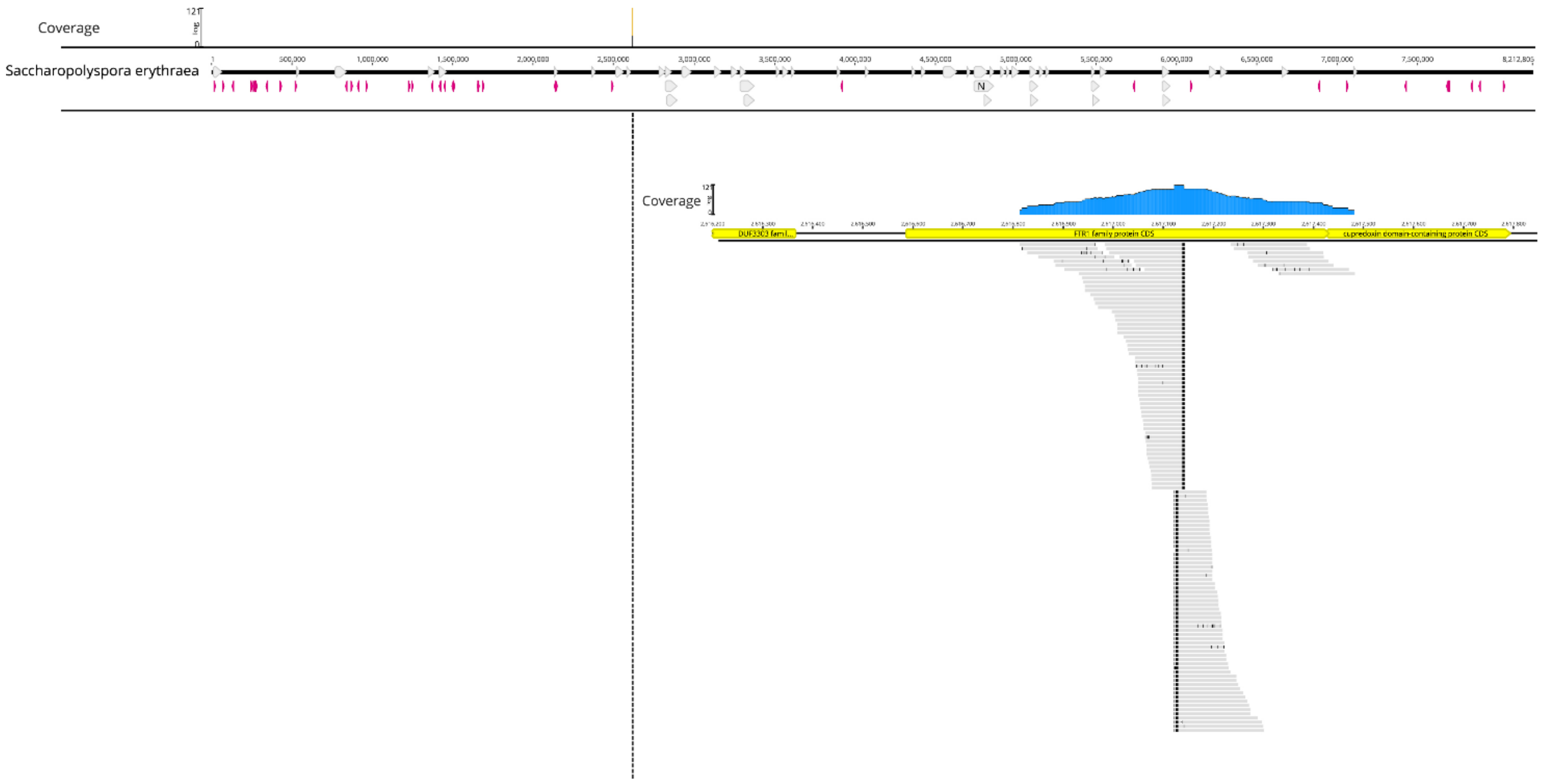

Fig. S5 Mapped reads retrieved with pRT801-attR/L probes to the genome sequence of *Sacch. erythraea.* All reads were aligned to the same region of the genome which was identified as a φBT1 *attB* site by chromosome retrieving approach. A close-view of the region is shown. Grey arrows represent BGCs, pink arrows – tRNA genes

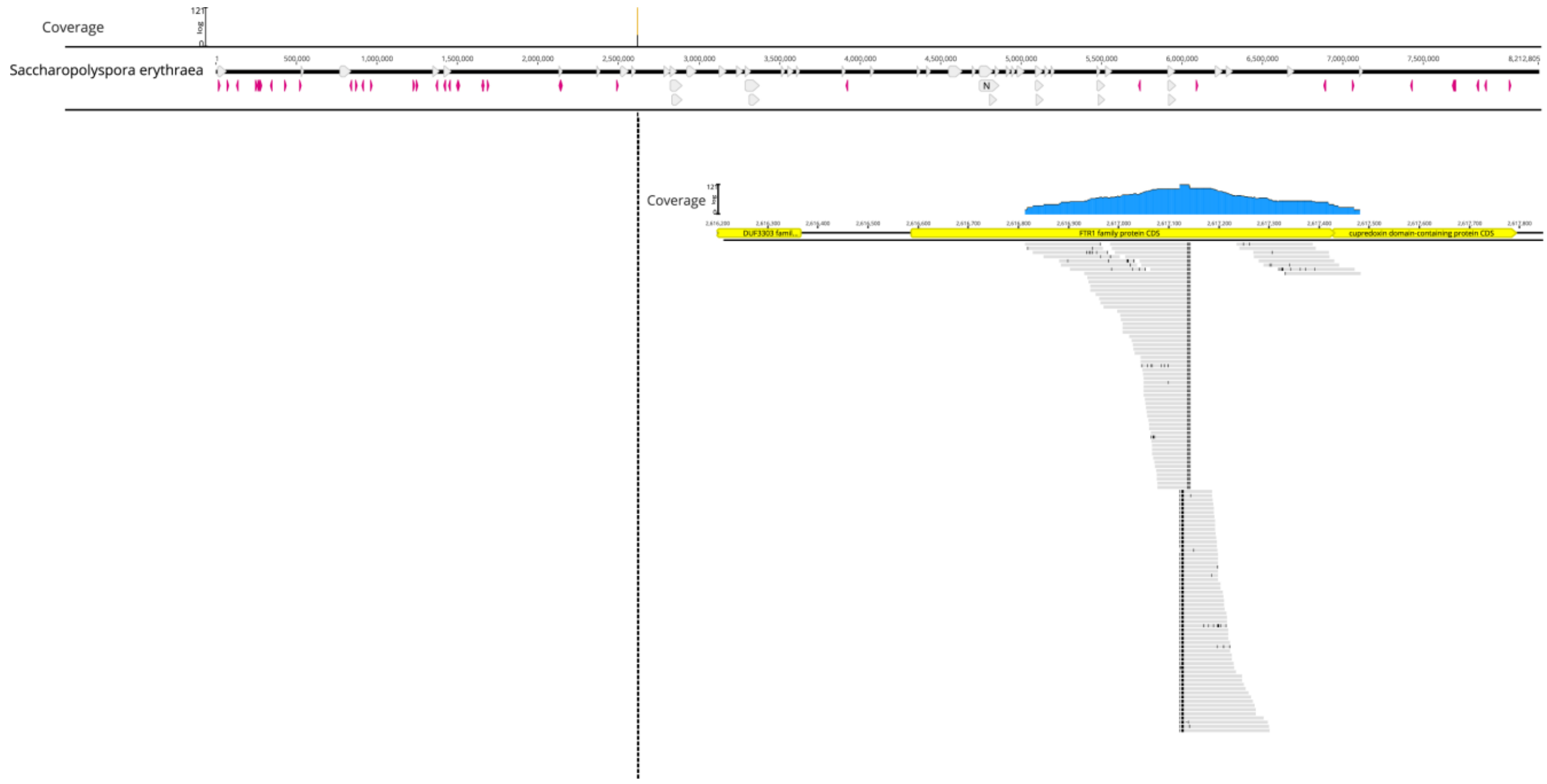

Fig. S6 Mapped reads retrieved with pCLY-attR/L probes to the genome sequence of *Sacch. erythraea.* All reads were aligned to the same region of the genome which was identified as a VWB *attB* site by chromosome retrieving approach. A close-view of the region is shown. Grey arrows represent BGCs, pink arrows – tRNA genes

pseudo-attB1

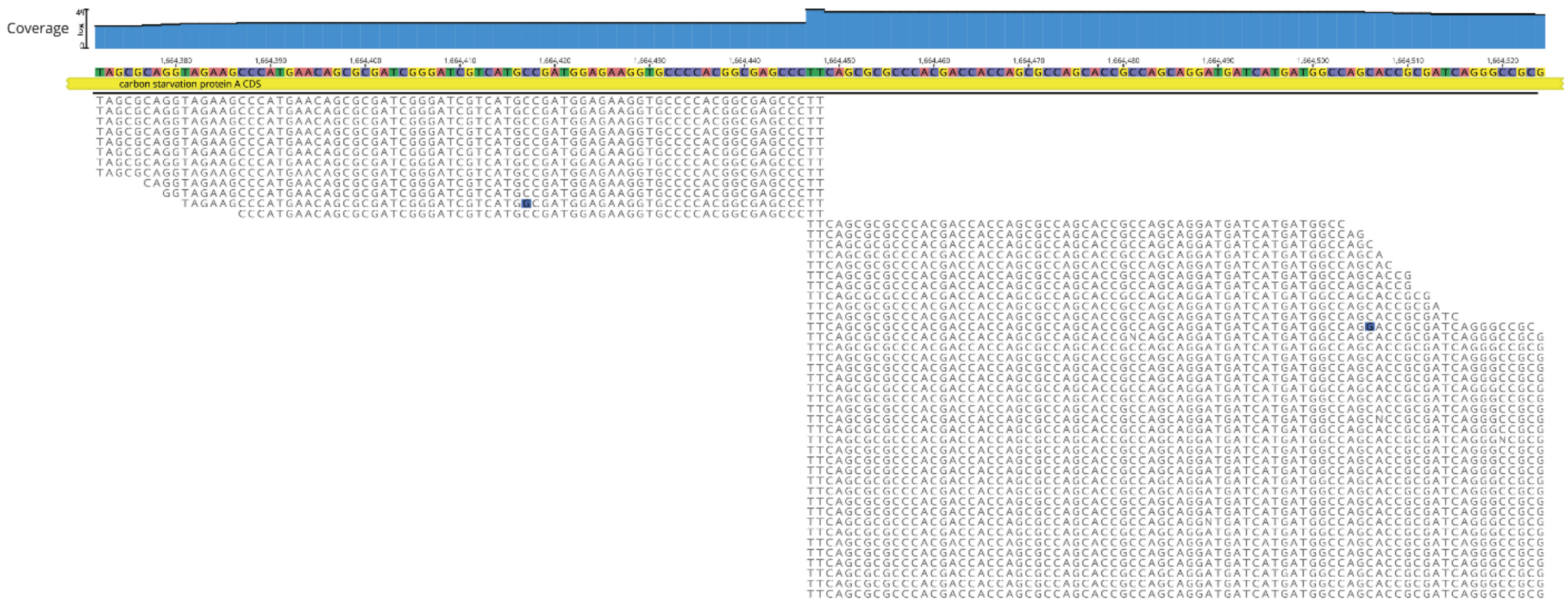

pseudo-attB2

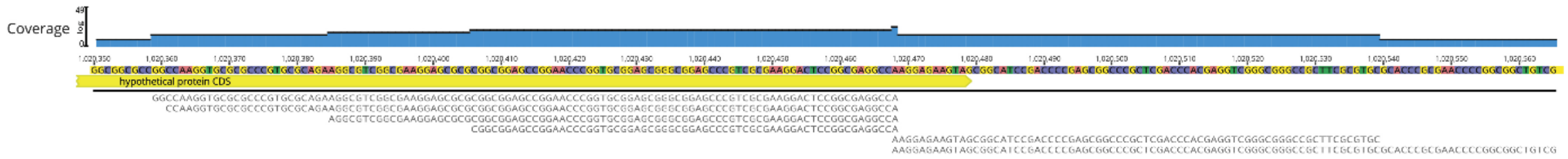

pseudo-attB3

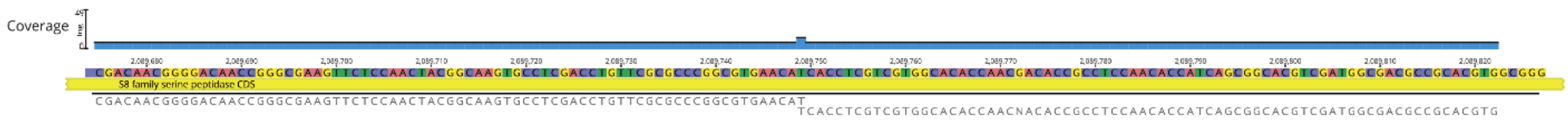

pseudo-attB4

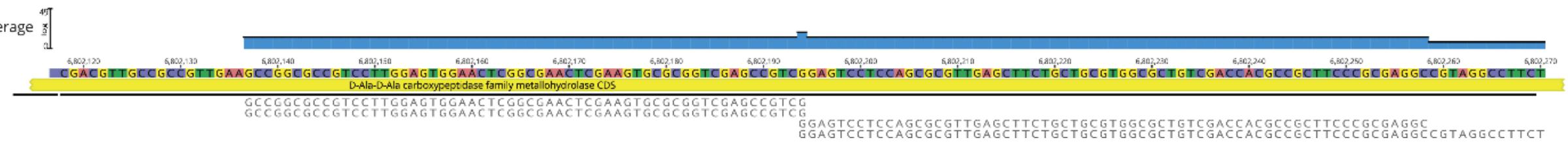

Fig. S7 Location of φC31 *attB* sites within the genome of *Sacch. erythraea*. Close view of regions of *Sacch. erythraea* genome with the mapped reads retrieved with pSET152-attR/L probes

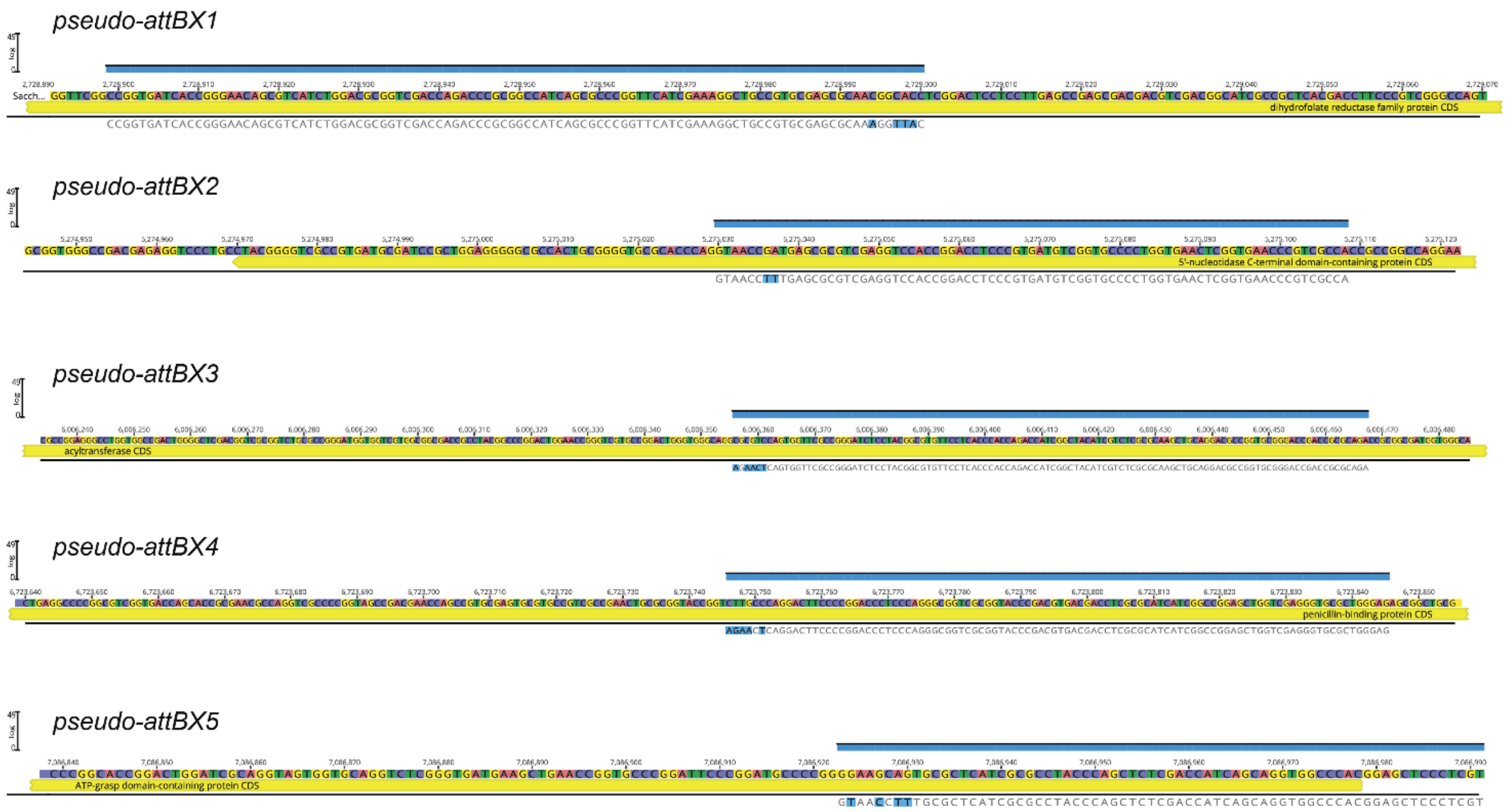

Fig. S8 Location of low efficiency φC31 *attB* sites within the genome of *Sacch. erythraea*. Close view of regions of *Sacch. erythraea* genome with the mapped reads retrieved with pSET152-attR/L probes

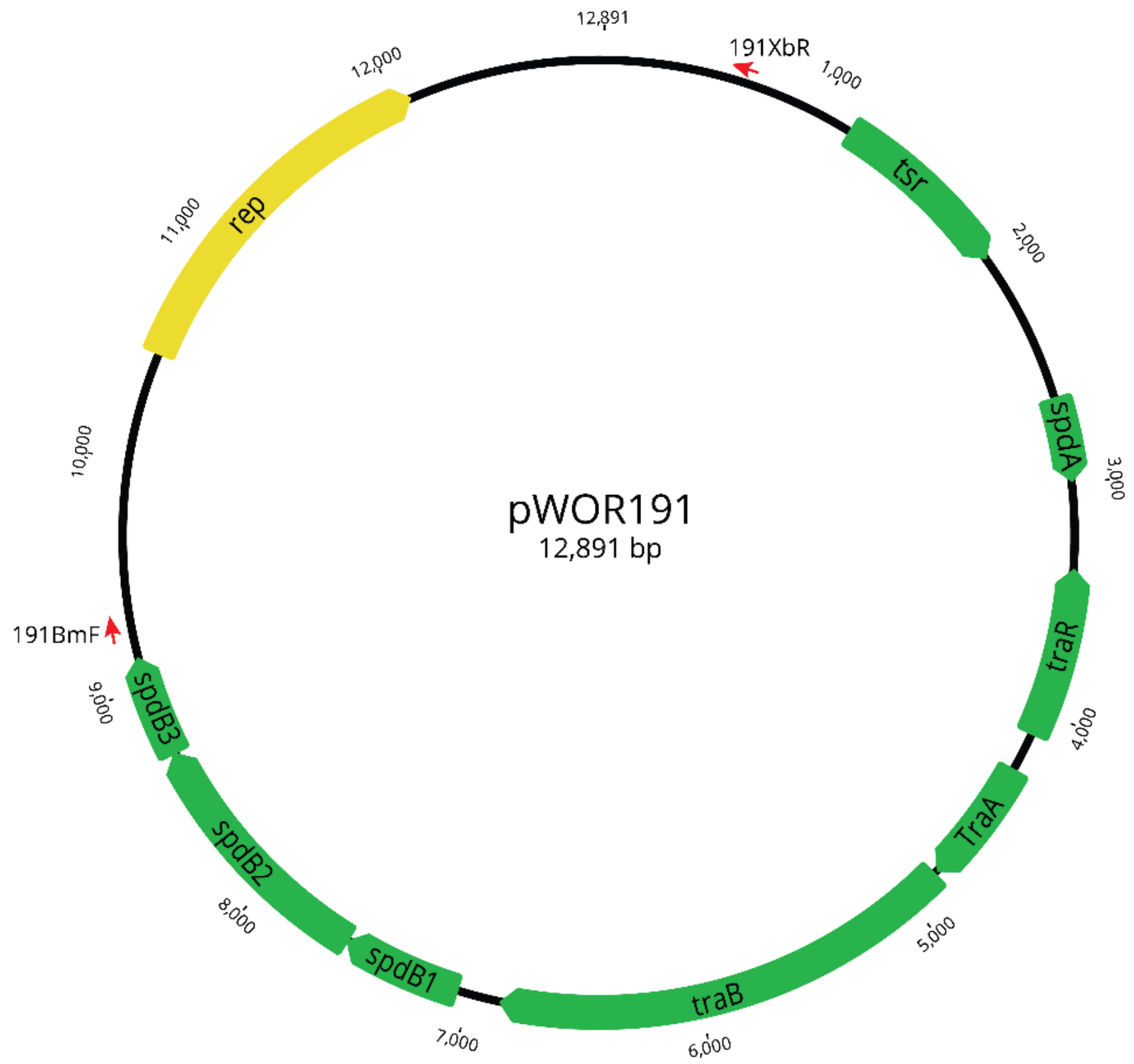

Fig. S9 Schematic representation of the pWOR191 plasmid. The plasmid contains the pJV1 replicon, including oriC, *rep*, *tra* (*tra*A, *tra*B, *tra*R), and *spd*B (*spd*B1, *spd*B2, *spd*B3) genes. For selection in actinobacteria the thiostrepton resistance gene was cloned (tsr) into pJV1

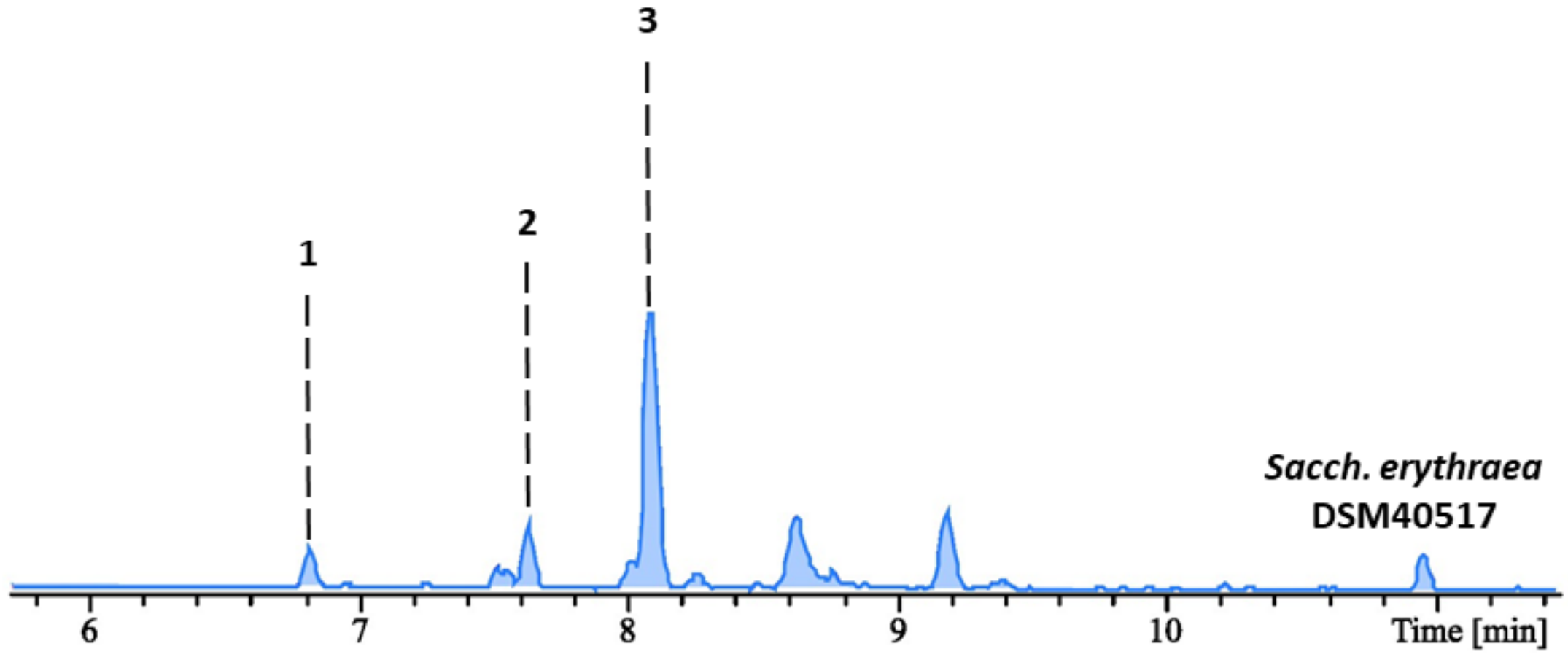

Fig. S10 Mass chromatograms of erythromycins with retention times (RT) from 6 to 9. 1 – Erythromycin A (RT 6.8 min, m/z = 734.46). 2 – Erythromycin B (RT 7.6, m/z = 718.46). 3 – Anhydroerythromycin A (RT 8.1, m/z = 716.45)

References:

Bierman, M., Logan, R., O'Brien, K., Seno, E. T., Rao, R. N., & Schoner, B. E. (1992). Plasmid cloning vectors for the conjugal transfer of DNA from Escherichia coli to Streptomyces spp. *Gene, 116*(1), 43-49. doi:10.1016/0378-1119(92)90627-2

Bilyk, O., Sekurova, O. N., Zotchev, S. B., & Luzhetskyy, A. (2016). Cloning and Heterologous Expression of the Grecocycline Biosynthetic Gene Cluster. *PLoS One, 11*(7), e0158682. doi:10.1371/journal.pone.0158682

Gregory, M. A., Till, R., & Smith, M. C. (2003). Integration site for Streptomyces phage phiBT1 and development of site-specific integrating vectors. *J Bacteriol, 185*(17), 5320-5323. doi:10.1128/JB.185.17.5320-5323.2003

Lal, P. B., Wells, F. M., Lyu, Y., Ghosh, I. N., Landick, R., & Kiley, P. J. (2019). A Markerless Method for Genome Engineering in Zymomonas mobilis ZM4. *Front Microbiol, 10*, 2216. doi:10.3389/fmicb.2019.02216

Myronovskyi, M., Rosenkranzer, B., & Luzhetskyy, A. (2014). Iterative marker excision system. *Appl Microbiol Biotechnol, 98*(10), 4557-4570. doi:10.1007/s00253-014-5523-z

Myronovskyi, M., Rosenkranzer, B., Nadmid, S., Pujic, P., Normand, P., & Luzhetskyy, A. (2018). Generation of a cluster-free Streptomyces albus chassis strains for improved heterologous expression of secondary metabolite clusters. *Metab Eng, 49*, 316-324. doi:10.1016/j.ymben.2018.09.004

Myronovskyi, M., Welle, E., Fedorenko, V., & Luzhetskyy, A. (2011). Beta-glucuronidase as a sensitive and versatile reporter in actinomycetes. *Appl Environ Microbiol, 77*(15), 5370-5383. doi:10.1128/AEM.00434-11

Tu, Q., Yin, J., Fu, J., Herrmann, J., Li, Y., Yin, Y., Zhang, Y. (2016). Room temperature electrocompetent bacterial cells improve DNA transformation and recombineering efficiency. *Sci Rep, 6*, 24648. doi:10.1038/srep24648
